# Biosynthesis, Regulation, and Biotechnological Production Strategies of Riboflavin (Vitamin B_2_) and Its Derivatives: A Review

**DOI:** 10.3390/ph19030389

**Published:** 2026-02-28

**Authors:** Raziel Arturo Jiménez-Nava, Griselda Ma. Chávez-Camarillo, Eliseo Cristiani-Urbina

**Affiliations:** 1 Departamento de Ingeniería Bioquímica, Escuela Nacional de Ciencias Biológicas, Instituto Politécnico Nacional, Unidad Profesional Adolfo López Mateos, Avenida Wilfrido Massieu s/n, Ciudad de México 07738, Mexico; rjimenezn@ipn.mx; 2 Departamento de Microbiología, Escuela Nacional de Ciencias Biológicas, Instituto Politécnico Nacional, Prolongación de Carpio y Plan de Ayala s/n, Colonia Santo Tomás, Ciudad de México 11340, Mexico

**Keywords:** riboflavin, vitamin B_2_, riboflavin biosynthetic pathway, strain improvement, microbial biotechnology, microbial fermentation

## Abstract

Riboflavin (RF; vitamin B_2_) is an essential micronutrient with broad applications in the food, feed, pharmaceutical, and cosmetic industries and is increasingly relevant in bioelectrochemical systems and environmental biotechnology. Microbial fermentation has replaced chemical synthesis as the dominant industrial production route due to its superior sustainability and scalability. However, despite substantial progress, RF biosynthesis remains constrained by imbalances in precursor supply, complex redox regulation, and regulatory feedback mechanisms that limit metabolic flux toward guanosine triphosphate and ribulose-5-phosphate. This review provides an updated, integrative analysis of RF biotechnology, encompassing biosynthetic pathways, transcriptional and redox-regulation, strain improvement strategies, and fermentation process optimization. Representative industrial producers—including *Bacillus subtilis*, *Ashbya gossypii*, and *Candida famata*—are critically evaluated for productivity, yield, and metabolic robustness, with reported titers reaching up to 29 g L^−1^ in engineered systems. Emerging microbial platforms, including lactic acid bacteria, thermotolerant and methylotrophic microorganisms, and electroactive bacteria, are discussed in the context of niche applications such as food biofortification and microbial fuel cells. Special emphasis is placed on oxidative stress as a regulatory signal influencing RF overproduction, metabolic rewiring strategies to alleviate precursor bottlenecks, and the biosynthesis of RF derivatives (FMN, FAD, roseoflavin, and 8-aminoriboflavin). In addition, biosafety, regulatory constraints, concerns about genome stability, and antibiotic-free engineering approaches are examined as critical determinants of future industrial competitiveness. By integrating molecular regulation, metabolic engineering, fermentation design, emerging applications, and regulatory perspectives within a unified framework, this review outlines current bottlenecks and future directions for developing safer, more robust, and economically competitive RF-producing microbial platforms.

## 1. Introduction

Riboflavin (RF), commonly known as vitamin B_2_, is a water-soluble B-complex vitamin. It is a natural, yellow, and fluorescent organic compound biosynthesized exclusively by plants and microorganisms; however, it is essential for all living organisms [1,2,3,4]. RF is the structural base of the flavin mononucleotide (FMN) and flavin adenine dinucleotide (FAD) coenzymes, which are essential electron carriers in the redox (reduction–oxidation) reactions of numerous metabolic pathways, including those involved in carbohydrate, lipid, amino acid, and protein metabolism; mitochondrial energy production (electron transport chain or respiratory chain); cellular homeostasis; and cellular antioxidant protection [5]. RF also plays a crucial role in the assimilation of iron and zinc and in the metabolism of vitamins B_3_ (niacin), B_6_ (pyridoxine), and B_9_ (folate) as well as related one-carbon (1C) compounds [1,3,5,6,7,8,9,10,11]. Additionally, RF is a structural component of cryptochromes (a group of blue light-sensing flavoproteins found in animals, plants, bacteria, and fungi), which are directly responsible for the downstream effects of light stimulation and, consequently, mediate various light-dependent cellular responses [12,13,14,15]. In nature, RF exists as free RF, RF glycosides, RF esters, and flavinyl peptides [3,4,16].

The recommended daily dietary intake of RF for healthy, well-nourished human adults ranges from 0.9 to 2.0 mg [1,16,17,18,19,20], which can be met by consuming various foods, including beef, veal, lamb, pork, fish, chicken, turkey, beef and veal liver and kidneys, eggs, milk, cheese, yogurt, broccoli, peanuts, soybean, kale, turnip, almond, edible mushrooms, green beans, spinach, asparagus, and wheat bran, or through dietary supplements [1,3,7,20,21,22].

Dietary riboflavin deficiency, also known as ariboflavinosis, remains prevalent in underdeveloped countries, primarily because of the insufficient intake of RF-rich foods [23]. Although ariboflavinosis is generally considered to be controlled in industrialized countries, certain population groups remain at risk. These include individuals with inadequate nutrition, older adults, those with increased nutritional requirements during pregnancy and lactation, and patients with hypothyroidism, diabetes mellitus, and chronic alcoholism [7,23]. Clinically, ariboflavinosis is associated with a broad spectrum of manifestations, including angular stomatitis, glossitis, cheilitis, seborrheic dermatitis, hair loss, growth retardation, mitochondrial dysfunction, corneal vascularization, edema of the pharyngeal and oral mucosa, photophobia, cataracts, hyperemia, renal damage, neurodegenerative alterations, and, in severe cases, hemolytic anemia. Some of these conditions are directly caused by RF deficiency, whereas others may arise from deficiencies in additional nutrients involved in RF metabolism [2,3,7,24].

In addition to dietary deficiency, RF transporter deficiency (RTD), also known as Fazio–Londe syndrome (FSL) or Brown–Vialetto–Van Laere syndrome (BVVLS), is a rare, life-threatening, autosomal recessive, and progressive neurodegenerative disorder affecting nerve cells in the brainstem and spinal cord, leading to sensorineural hearing loss, sensory ataxia, cranial nerve palsies, respiratory dysfunction, optic atrophy, progressive muscle weakness, dysphagia, and dysarthria [25,26,27]. RTD typically manifests during infancy, childhood, or adolescence. Its diagnosis is often difficult owing to its rarity, with an estimated incidence of approximately 70 reported cases per year. However, most of the affected individuals remain undiagnosed throughout their lives. RTD is caused by mutations in *SLC52A2* and *SLC52A3*, which encode the RF transporters RFVT2 and RFVT3, respectively. RFVT3 is the principal component responsible for intestinal RF absorption, whereas RFVT2 primarily mediates intracellular and inter-tissue RF transport, highlighting the physiological relevance of these transporters [25,26,27].

Both ariboflavinosis and RTD are managed clinically with oral RF supplementation at high doses, generally ranging from 0.6 to 80 mg/kg/day or from 200 to 1800 mg/day, where affected patients require at least the minimum doses (0.6 mg/kg/day or 200 mg/day) to achieve measurable clinical improvements [28].

In addition to preventing the common symptoms of RF deficiency, RF intake improves a wide variety of physiological health functions [10,29] as it functions as a natural antioxidant [8,10], photosensitizer [30], and neuroprotective agent [29]. RF has been shown to protect cells from lipid peroxidation, oxidative DNA damage, and protein carbonyl accumulation [8,10]. Likewise, RF reduces the risk of cataract formation [31] and osteoporosis [32,33], modulates malaria infection [34], strengthens the immune system [20,35,36,37,38], reduces the frequency and severity of migraines [39,40], reduces the risk and acts as a therapeutic treatment agent for certain cancers [29,41], possesses anti-inflammatory and antinociceptive effects [42,43,44,45,46], decreases premenstrual syndrome [47,48], attenuates oxidative injuries [29], prevents and ameliorates normochromic and normocytic anemia [49,50], participates in corneal cross-linking [51], and contributes to the prevention of root caries [52], among other beneficial effects [10,53,54,55,56]. Furthermore, the photochemical and antioxidant properties of RF are currently being exploited to inhibit pathogenic microorganisms and extend the shelf life of foods [57,58,59,60,61,62].

Although RF can be synthesized chemically and chemoenzymatically, or extracted from plant and animal tissues, its industrial production almost exclusively relies on microbial fermentation because of its efficiency, cost-effectiveness, and low environmental impact. The industrial microbial production of RF is considered a brilliant advance in industrial biotechnology, successfully replacing traditional synthetic chemical processes [6,11,18,63,64,65,66].

Over 12,700 tons of RF are currently produced worldwide annually through microbial fermentation using engineered strains of *Bacillus subtilis*, *Eremothecium ashbyi*, and *Ashbya gossypii* (heterotypic synonym for *Eremothecium gossypii*) [66,67,68,69], with Hubei Guangji Pharmaceuticals (Huanggang, China), Shanghai Acebright Pharmaceuticals Group (Shanghai, China), Henan Julong Biological Engineering (Ruzhou, China), BASF (Ludwigshafen, Germany and Seoul, Republic of Korea), and DSM-Firmenich (Leeuwarden, The Netherlands) being the major RF producers globally [6,9,22,66,68,69]. The RF market size was estimated at USD 451.47 million in 2024 and is expected to reach USD 623.06 million by 2029, growing at a compound annual growth rate (CAGR) of 6.65% during the period 2024–2029 [9,69,70]. Furthermore, the European Food Safety Authority (EFSA) Panel on Additives and Products or Substances used in Animal Feed (FEEDAP) recently concluded (2024) that the use of a feed additive containing at least 5% RF produced using the filamentous fungus *Eremothecium ashbyi* CCTCCM 2019883 by Hubei Guangji Pharmaceutical Co. is of no safety concern for all animal species, supporting the use of this filamentous fungus in commercial large-scale RF fermentation processes [71,72].

Approximately 70% of the current global RF production is used as a livestock nutritional supplement—principally for swine [73] and poultry [74,75,76,77,78,79,80,81,82,83,84]—whereas 10–20% is utilized as a natural yellow food and beverage colorant (E-101), and approximately 10% is used in cosmetic and pharmaceutical applications [11,66,67,85].

Despite the increasing diversity of RF-producing microorganisms, only a limited number of strains currently exhibit productivities and yields compatible with industrial-scale manufacturing. Engineered strains of *Bacillus subtilis*, *Ashbya gossypii*, and *Candida famata* consistently achieve titters in the range of 13–29 g L^−1^, with volumetric productivities reaching up to ~0.6 g L^−1^ h^−1^ under optimized conditions [86,87,88]. These metrics clearly position them as competitive industrial platforms for bulk RF production. In contrast, lactic acid bacteria and other moderate producers, although physiologically capable of RF overproduction, generally operate within the milligram-per-liter range and are therefore better suited for food biofortification and functional fermentation applications rather than commodity-scale production [89].

Nevertheless, several other bacteria, yeasts, and filamentous fungi possess physiological traits that make them promising candidates for large-scale RF production in future. Recent reviews have summarized advances in microbial RF production up to the last five years [6,11,20,66,90], or have focused on specific microorganisms, such as *Bacillus subtilis*, *A. gossypii*, and *Candida famata* [69,91,92] or on molecular tools applicable to yeasts and filamentous fungi [9]. However, limited information is available regarding the advantages and potential of emerging RF-producing microorganisms. Although these novel microbial species currently produce lower RF titers than those obtained with industrial *B. subtilis* and *A. gossipy* strains, they exhibit valuable attributes, including thermotolerance, ability to grow and produce RF from agro-industrial wastes or by-products, non-food substrates, non-feed substrates, or culture media with low organic content, which position them as potential alternatives for RF production [68,93,94,95,96,97,98,99].

While several recent reviews have addressed advances in microbial RF production—often focusing on specific industrial hosts, metabolic engineering tools, or process optimization strategies—the present review aims to provide an integrative and updated perspective that critically examines regulatory mechanisms, redox-associated control, precursor supply complexity, emerging microbial platforms, RF derivatives, and biosafety considerations within a unified framework.

This review summarizes the current knowledge on microbial RF biosynthesis and its regulatory mechanisms, along with strategies to enhance RF and RF-precursor production through optimization of fermentation culture conditions and the development of RF-overproducing microbial strains through chemical mutagenesis and metabolic engineering. Additionally, it highlights recent developments in RF-enriched foods and beverages, microbial fuels produced by electroactive bacteria for renewable energy and environmental remediation, and the microbial production of RF derivatives.

## 2. The RF Biosynthetic Pathway in Microorganisms

**Figure 1:**
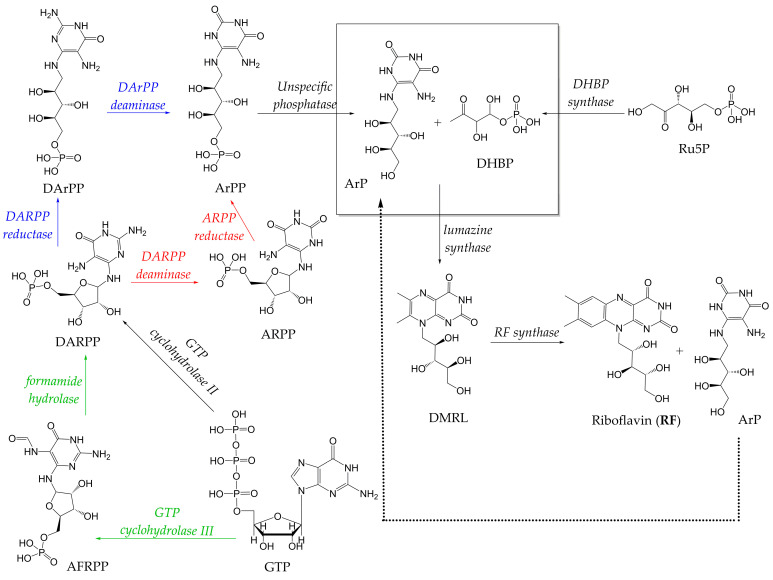
The riboflavin biosynthetic pathway (RFBP). GTP, guanosine triphosphate; DARPP, 2,5-diamino-6-ribosyl-amino-4(3*H*)-pyrimidinone 5′-phosphate; DArPP, 2,5-diamino-6-ribityl-amino-4(3*H*)-pyrimidinone 5′-phosphate; ARPP, 5-amino-6-ribosyl-amino-2,4(1*H*,3*H*)-pyrimidinedione 5′-phosphate; ArPP, 5-amino-6-ribityl-amino-2,4(1*H*,3*H*)-pyrimidinedione 5′-phosphate; ArP, 5-amino-6-ribityl-amino-2,4(1*H*,3*H*)-pyrimidinedione; Ru5P, ribulose-5-phosphate; DHBP, 3,4-dihydroxy-2-butanone-4-phosphate; DMRL, 6,7-dimethyl-8-ribityllumazine; AFRPP, 2-amino-5-formylamino-6-ribosylamino-4(3*H*)-pyrimidinone 5′-phosphate. In plants and eubacteria, the deamination reaction followed by the reduction reaction is shown in red; in archaea, yeasts, and filamentous fungi, the reduction reaction followed by the deamination reaction is shown in blue; and the alternative pathway described in several archaea and in *Sinorhizobium meliloti* is shown in green. The figure was constructed based on information from references [6,11,17,22,90,106]. All structures were generated using the PubChem database to ensure chemical accuracy and consistent graphical representation.

The RF biosynthetic pathway (RFBP) comprises seven enzymatic reactions that convert precursor molecules into RF (Figure 1). The structural precursors of RF biosynthesis are two molecules of ribulose-5-phosphate (Ru5P) and one molecule of guanosine triphosphate (GTP), which together result in the biosynthesis of one RF molecule [17,22,100,101,102]. Both Ru5P and GTP are derived from central carbon metabolism; Ru5P originates from the pentose phosphate pathway (PPP), while GTP is synthesized through the purine biosynthetic pathway (PBP) [100,103,104,105].

The first step in the RFBP is the hydrolysis of the GTP imidazole ring catalyzed by the GTP cyclohydrolase II to produce 2,5-diamino-6-ribosyl-amino-4(3*H*)-pyrimidinone 5′-phosphate (DARPP) [6,17,20,22,90]. The sequence of subsequent reactions varies depending on the organism involved. In plants and eubacteria, DARPP is first deaminated to 5-amino-6-ribosyl-amino-2,4(1*H*,3*H*)-pyrimidinedione 5′-phosphate (ARPP), which is then reduced to 5-amino-6-ribityl-amino-2,4(1*H*,3*H*)-pyrimidinedione 5′-phosphate (ArPP). In contrast, in archaea, yeasts, and filamentous fungi, DARPP is first reduced to 2,5-diamino-6-ribityl-amino-4(3*H*)-pyrimidinone 5′-phosphate (DArPP), which is then deaminated to ArPP [6,11,100,107,108].

The fourth step involves ArPP dephosphorylation to produce 5-amino-6-ribityl-amino-2,4(1*H*,3*H*)-pyrimidinedione (ArP) [90]. Until recently, the mechanisms underlying ArPP dephosphorylation remained unclear [90]. Current evidence from plant and microbial physiology indicates that several enzymes from the haloacid dehalogenase (HAD) superfamily catalyze this reaction [109,110,111,112,113]. Most HAD enzymes also exhibit FMN phosphatase activity [104,107,108]. Furthermore, deletion of individual ArPP phosphatase-encoding genes does not result in RF auxotrophy, suggesting the existence of several isoenzymes [112,114]. Notably, overaccumulation of the intermediate ArP can cause growth impairment [88].

Recently, a specific ArPP phosphatase was described in the chemolithoautotrophic bacterium, *Aquifex aeolicus*. This novel enzyme belongs to the histidine phosphatase superfamily and has a structure resolved at 2.04 Å, a molecular mass of approximately 22 kDa, and 812 amino acid residues. In addition to dephosphorylating ArPP, this enzyme exhibits D-ribose 5-phosphate (R5P) isomerase activity, catalyzing the reversible interconversion of R5P and Ru5P. Both enzyme activities may enhance carbon flux through the RFBP [115].

The fifth reaction involves Ru5P conversion to 3,4-dihydroxy-2-butanone-4-phosphate (DHBP), catalyzed by DHBP synthase. In plants and several Gram-positive bacteria such as *B. subtilis*, DHBP synthesis and GTP hydrolysis co-occur through the bifunctional enzyme GTP cyclohydrolase II/DHBP synthase, encoded by the *ribA* gene. The DHBP synthase and GTP cyclohydrolase II domains reside in the N-terminal- and C-terminal regions of the protein, respectively [6,17].

In the sixth reaction, lumazine synthase catalyzes the condensation of DHBP and ArP to form 6,7-dimethyl-8-ribityllumazine (DMRL). Finally, RF synthase converts two molecules of DMRL into one molecule of RF and one molecule of ArP, which is then recycled back into the RFBP [6,11,20,22,90].

The RFBP of archaea differs from that of bacteria in the initial steps of the pathway, as illustrated by the green lines in Figure 1. Bacteria initiate RFBP with GTP cyclohydrolase II, forming DARPP from GTP, whereas archaea utilize GTP cyclohydrolase III, which converts GTP into 2-amino-5-formylamino-6-ribosylamino-4(3*H*)-pyrimidinone 5′-phosphate (AFRPP). AFRPP is subsequently deformylated by formamide hydrolase to form DARPP. The remaining steps are conserved in both bacteria and archaea [17,116]. This RFBP has been described in the archaeal species *Methanocaldococcus jannaschii* [112] and, more recently, in the bacterium *Sinorhizobium meliloti* [106]. In the latter, the SMc02977 protein has been annotated as a formamide hydrolase, with homologs identified in several invasive bacterial taxa, including *Clostridium* spp., *Brucella abortus*, *Mycobacterium tuberculosis*, *Listeria monocytogenes*, and *Ochrobactrum anthropi* [106].

Interestingly, some bacteria such as *Sinorhizobium meliloti* have two distinct routes of RF production: the well-known pathway (beginning with GTP hydrolysis, followed by deamination and reduction reactions) [117] and an alternative formamidase pathway involving formamide hydrolase [106].

Bacteria can also acquire RF through overlapping strategies, including de novo biosynthesis and uptake via importer proteins. These mechanisms are not mutually exclusive; many bacteria have multiple paralogs of biosynthetic enzymes and transporters, suggesting a flexible regulatory network that enables bacterial cells to switch between RF import and biosynthesis depending on the environmental conditions. Similarly, the expression of RF biosynthesis and transport genes is highly regulated by several factors [118].

## 3. The Complexity of Precursor Supply for RF Biosynthesis

Although RFBP is a specific metabolic process, several non-specific metabolic reactions are involved in the metabolic flux of carbon and nitrogen toward the RF precursors GTP and Ru5P. Moreover, RF precursor availability is governed by multiple interconnected, tightly regulated metabolic routes, as these precursors are not freely available, but arise from other complex biosynthetic processes. This interconnectedness makes the overall supply network for RF biosynthesis highly intricate [20,100,119,120,121].

Although bacteria and yeasts can biosynthesize RF precursors from glucose and organic or inorganic nitrogen sources, filamentous fungal strains preferentially utilize vegetable oils such as rapeseed, sunflower, corn, soybean, and waste cooking oils as fermentation substrates [6,22,100,122,123,124].

**Figure 2:**
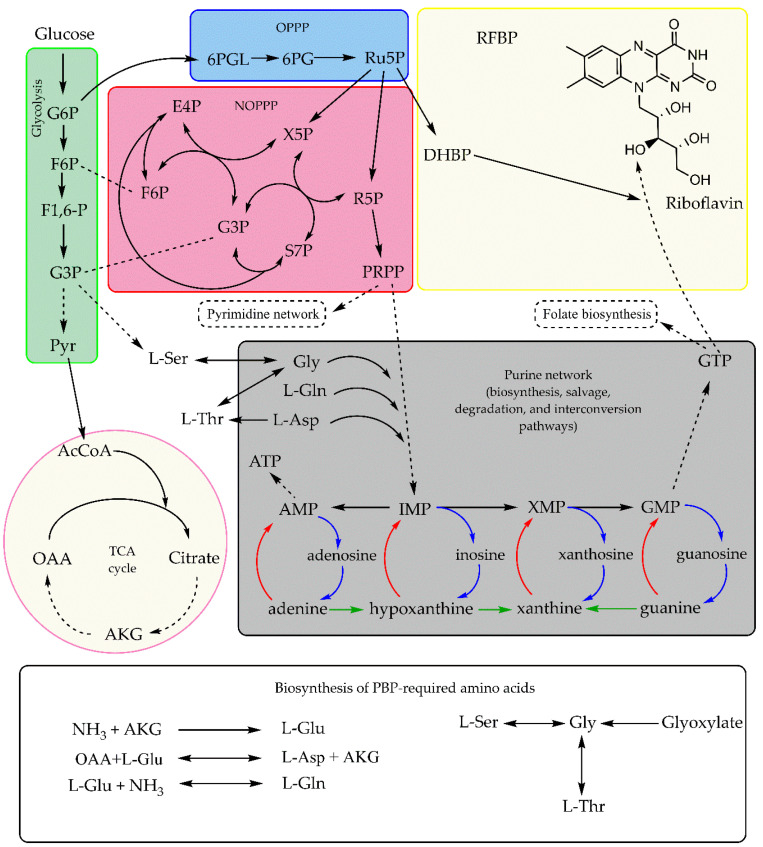
The riboflavin biosynthetic pathway (RFBP, yellow box) and interconnected metabolic pathways in microorganisms. Glycolysis (green box); non-oxidative pentose phosphate pathway (NOPPP, red box); oxidative pentose phosphate pathway (OPPP, blue box); tricarboxylic acid (TCA) cycle (pink circle); purine network, including biosynthesis, salvage, degradation, and interconversion pathways (gray box); biosynthesis of amino acids required for the PBP (white box). The dashed white boxes represent the pyrimidine network and folate biosynthesis pathways, which are metabolically competitive with RF biosynthesis because they share key cofactors and intermediates. G6P, glucose-6-phosphate; F6P, fructose-6-phosphate; F1,6-P, fructose-1,6-biphosphate; G3P, glyceraldehyde-3-phosphate; Pyr, pyruvate; AcCoA, acetyl-CoA; OAA, oxalacetate; AKG, α-ketoglutarate; TCA cycle, tricarboxylic acid cycle; 6PGL, 6-phosphogluconolactone; 6PG, 6-phosphogluconate; Ru5P, ribulose-5-phosphate; R5P, ribose-5-phosphate; X5P, xylulose-5-phosphate; E4P, erythrose-4-phosphate; S7P, sedoheptulose-7-phosphate; PRPP, phosphoribosyl pyrophosphate; Gly, glycine; L-Gln, L-glutamine; L-Asp, L-aspartate; AMP, adenosine monophosphate; IMP, inosine monophosphate; XMP, xanthosine monophosphate; GMP, guanosine monophosphate; ATP, adenosine triphosphate; GTP, guanosine triphosphate; DHBP, 3,4-dihydroxy-2-butanone-4-phosphate; NH_3_, ammonia; L-Ser, L-serine; L-Thr, L-threonine; L-Glu, L-glutamate. Dotted arrows represent abbreviated reactions; solid arrows denote direct reactions. Green arrows denote purine interconversion reactions, red arrows indicate purine salvage reactions, and blue arrows represent purine nucleotide and nucleoside degradation reactions. The figure was constructed based on information from references [70,105,126,127,128,129].

RF precursor biosynthesis in bacteria and yeasts is relatively direct. Glucose enters the oxidative pentose phosphate pathway (OPPP) as glucose-6-phosphate, producing phosphoribosyl pyrophosphate (PRPP) and Ru5P, the former being the initial substrate for the purine biosynthetic pathway (PBP) [22]. However, in filamentous fungi, RF precursor biosynthesis is more complex. Acetyl-CoA, originating from fatty acid β-oxidation, is directed to the glyoxylate cycle, where malate production influences the PBP [100,125]. Subsequently, the carbon flux is channeled into gluconeogenesis and finally fed into the PPP [17,20,22,100,122]. This complex interplay between RF biosynthesis and other cellular metabolic pathways is illustrated in Figure 2.

Both the non-oxidative and oxidative branches of the PPP are essential to microbial cellular metabolism, as their intermediates and products are required for the biosynthesis of NADPH, pyrimidines, purines, aromatic amino acids, carotenoids, polyols, shikimic acid, antibiotics, as well as vitamins K, B_6_, and B_9_ [104,105,108]. Among these carbon intermediates, PRPP and Ru5P are key precursors for RF biosynthesis [17,22].

PPP intermediates, such as sedoheptulose-7-phosphate (S7P), ribose-5-phosphate (R5P), and xylulose-5-phosphate (X5P), can redirect carbon flux back into the glycolysis pathway, negatively affecting RF biosynthesis. Consequently, mutations that disrupt or attenuate the expression of genes encoding the enzymes involved in S7P and X5P biosynthesis have been shown to increase RF production [70,126]. Furthermore, the overexpression of PPP enzymes, such as 6-phosphogluconate dehydrogenase [130,131], glucose-6-phosphate dehydrogenase [132,133], 6-phosphogluconolactonase [134], ribose-5-phosphate isomerase [132], and fructose-1,6-bisphosphatase [68], has proven effective in directing carbon flux toward PRPP and Ru5P.

Another notable approach involves engineering microbial strains capable of using alternative substrates, such as xylose, mannitol, spent sulfite liquor, hydrolysates of corn cob, sugarcane bagasse, xylose-rich rice husk, and mannitol-rich brown seaweed [68,94,96,135,136], as well as gluconate [137]. These substrates reduce the carbon flux through glycolysis and enhance RF biosynthesis.

With respect to GTP biosynthesis, purine metabolism represents an exceptionally complex network involving degradation of purine nucleotides to purine nucleosides and, subsequently, to purine bases; purine base interconversion pathways; and purine nucleotide synthesis via different purine phosphoribosyltransferases (PRTs) through the purine salvage pathway (Figure 2). Furthermore, purine biosynthesis is subject to feedback inhibition by adenosine and guanosine mono-, di-, and triphosphates (AMP, ADP, ATP, GMP, GDP, and GTP). These regulatory mechanisms can limit the carbon flux required to enhance RF production, because RF biosynthesis competes with adenosine metabolism [70,95,127].

Upregulation of purine biosynthetic genes has been shown to be effective at counteracting feedback inhibition within the PBP in *Escherichia coli* and *B. subtilis* [138,139,140]. In *A. gossypii*, overexpression or enzyme engineering to replace specific amino acid residues of PRPP synthase and PRPP amidotransferase reduces feedback inhibition by ADP, ATP, and GTP [141,142]. However, simultaneous enzyme engineering and overexpression of genes encoding PRPP synthase (*PRS3*) and PRPP amidotransferase (*ADE4*) have proven beneficial only in *C. famata*, where they increase precursor availability for RF biosynthesis and ultimately increase RF production [102].

GTP biosynthesis requires the amino acids glycine, L-glutamine, and L-aspartate [103,143,144], which can be exogenously supplied to the growing culture medium or synthesized endogenously from ammonia (NH_3_), L-glutamate [128], and various central carbon intermediates such as α-ketoglutarate, oxalacetate, 3-phosphoglycerate, glyoxylate, L-serine, and L-threonine [100,128,129,143,144,145,146]. As these carbon intermediates participate in other central metabolic pathways and are influenced by carbon assimilation and transport processes, their strict regulation is essential to meet metabolic demands [144].

Glycine is commonly added to culture media to increase microbial RF production [18,22,100,121,147,148,149,150,151,152]. Although its supplementation increases production costs, direct glycine assimilation is metabolically less expensive for microbial cells than its biosynthesis from carbon intermediates because glycine biosynthesis requires more enzymatic steps than L-glutamine or L-aspartate biosynthesis [100,128,129]. This reduced synthesis burden results in increased RF production.

Additionally, both the folate biosynthesis pathway and the pyrimidine network can compete metabolically with the RF biosynthesis pathway. Both folate and RF biosynthesis require GTP as the starting substrate, resulting in competition at an early metabolic step [17,124,153]. PRPP, in turn, is a crucial precursor for both purine and pyrimidine biosynthesis, serving as an activated form of R5P, an essential intermediate in de novo biosynthetic pathways and in salvage routes of nucleotide metabolism [104]. Therefore, metabolic engineering approaches aimed at reducing carbon flux into pyrimidine biosynthesis have also been successful at enhancing RF production [134].

As modifications to the PPP and purine and pyrimidine networks to enhance the production of the precursors Ru5P and GTP, and consequently RF, can lead to impaired growth or accumulation of the toxic intermediate ArP, these engineering efforts must be implemented cautiously [70,88,95,99,126]. Nonetheless, rational engineering strategies targeting the PPP and PBP have proven successful in certain microorganisms and have improved RF production [70,95,132,133,134,137].

## 4. Fermentation Operation Modes for RF Production and the Relationship Between Microbial Growth and RF Production

The most common fermentation modes used for RF production are the batch and fed-batch modes. Some RF-producing processes begin with batch fermentation to accumulate biomass, but frequently transition to the fed-batch mode to optimize nutrient availability and maximize RF production. Fed-batch fermentation is currently the preferred mode for large-scale RF production because it supports higher biomass densities, greater productivity of growth-related metabolites, reduced metabolic overflow and substrate inhibition, and improved control of abiotic and biotic parameters that influence RF biosynthesis [87,88,132,154,155,156,157,158]. Further, several studies have examined continuous fermentation as a means of optimizing specific operational variables, such as the dilution rate, and consequently maximizing steady-state RF production [152,159,160,161,162,163,164,165,166].

Although RF is an intermediate of primary metabolism [167,168], studies evaluating different fermentation modes have shown that RF biosynthesis in filamentous fungi is not directly linked to microbial growth but is instead decoupled from it [18,22]. In *A. gossypii*, RF production is correlated with culture aging, nutrient depletion, declining growth rates, septation within hyphae, vacuole enlargement, and sporulation onset [161,162,169,170,171]. Therefore, RF is considered a “pseudo-secondary metabolite” in *A. gossypii* [17].

In contrast, RF production by yeasts and bacteria in batch and continuous cultures indicates that RF behaves as a mixed-growth-associated metabolite [18,22,121,152,164]. In batch cultures, RF synthesis is absent during the lag and early exponential phases and reaches its maximum rate during the mid- and late-exponential growth phases [121]. In chemostat cultures, RF production is both growth-dependent and -independent, and is influenced by the specific growth rate (dilution rate) and biomass concentration. The Luedeking-Piret mathematical model accurately describes this dual behavior of product production and is widely applied to characterize mixed-growth-associated metabolites [152,164,172,173].

## 5. RF Production by Filamentous Fungi, Yeasts, and Bacteria: State of the Art in RF Biotechnology

A wide variety of microorganisms, including bacteria, yeasts, and filamentous fungi, can produce RF, and several species are used industrially. These include filamentous fungi, such as *Ashbya gossypii* and *Eremothecium ashbyii*, yeasts, such as *Candida famata*, and bacteria, such as *Bacillus subtilis* and *Corynebacterium ammoniagenes*.

RF biosynthesis is a highly regulated process in microorganisms to meet their metabolic needs as well as prevent harmful imbalances. Regulation occurs at the transcriptional, translational, and post-translational levels. Regulatory factors, such as carbon and nitrogen sources and metal ions (iron, chromium, zinc, cobalt, copper, and magnesium), also affect RF production. Consequently, several flavinogenic microorganisms have been extensively modified and engineered to achieve overproduction of RF.

Distinct RF-regulatory phenomena exist among microbial groups. In filamentous fungi, RF overproduction is primarily triggered by oxidative stress [161,162,174,175,176,177,178,179,180]. In yeasts, RF production is closely linked to iron assimilation regulation [17,167,181,182,183,184,185,186,187,188,189]. In bacteria, transcriptional and translational regulation is mainly controlled by conformational changes in the FMN riboswitch (*RFN* element) [6,11,22,85,90,91,190,191,192,193,194].

Following is an updated overview of RF production by flavinogenic microorganisms.

### 5.1. RF Production by Filamentous Fungi

#### 5.1.1. *Ashbya gossypii*: The Most Robust Natural RF Overproducer

The filamentous fungus *Ashbya gossypii* (a heterotypic synonym of *Eremothecium gossypii*) is a natural RF overproducer, and together with *B. subtilis*, represents the dominant microbial chassis for industrial RF production [6,22,90]. RF overproduction in *A. gossypii* was discovered by Wickerham et al. in 1946 [18,100,195]. In the 1960s, Merck commissioned Arnold Demain to develop a microbial RF production process using *A. gossypii* [18,85]. During the 1990s, BASF, together with researchers from the Jülich Institute for Biotechnology (H. Seulberger, H. Sahm, R. Krämer, and K.-P. Stahmann) and the University of Salamanca (J. L. Revuelta-Doval, R. Ledesma-Amaro, R. Martínez-Buey, and A. Jiménez-García) developed potent industrial RF-producing strains based on *A. gossypii* [85].

Engineered strains of *A. gossypii* commonly produce 15–20 g L^−1^ RF in 6–8-day fermentations under optimized fed-batch conditions [22,85,196,197,198,199]. In contrast, wild-type strains typically produce only 100–300 mg L^−1^ [180,200], highlighting the substantial impact of metabolic engineering and process optimization on industrial performance. A remarkable characteristic of this fungus is that its RF production is strongly induced by oxidative [174,175,176,180,200], nutritional [161], and endoplasmic reticulum (ER) stress [201].

*A. gossypii* is a natural phytopathogen found in tomato, coffee, citrus, and cotton crops [202]. It has been isolated from insects living on alkaloid-producing (milkweed and oleander) as well as non-alkaloid-producing plants (maple and boxelder). However, RF-overproducing *A. gossypii* isolates are associated with insects feeding on alkaloid-rich plants but not with those feeding on non-alkaloid-producing plants, suggesting that RF aids insects in alkaloid detoxification [177].

*A. gossypii* is an obligately aerobic filamentous fungus. Aerobic metabolism inevitably produces reactive oxygen species (ROS), which can damage cellular components but also function as critical signaling molecules [181,203,204,205,206]. Therefore, cells must maintain a balance between antioxidant defenses and ROS production to prevent oxidative stress [176,207]. RF acts as an antioxidant by enhancing the activity of enzymes such as catalase (CAT), superoxide dismutase (SOD), and glutathione reductase (GR), which are essential for ROS detoxification [8,208]. RF has been shown to protect cells from lipid peroxidation [54,209,210], oxidative DNA damage [46,56,211], and protein carbonyl accumulation [53,54,55]. Glutathione (GSH) is an antioxidant, and RF in its FAD form is required for the regeneration of GSH by GR to maintain redox homeostasis [8,208]. Additionally, the RF precursor DARPP protects *Saccharomyces cerevisiae* from nitrosative stress [212].

In *A. gossypii*, the transcription factor Yap1 plays a key role in oxidative stress response and is directly associated with RF overproduction. Yap1 activation upregulates the genes involved in stress tolerance and RF biosynthesis [176].

*A. gossypii* produces RF as a protective agent against UV radiation during its sporulation phase, demonstrated by the need to double the exposure time to UV radiation for completely inactivating fungal ascospores in the presence of RF [162].

Furthermore, intracellular ROS accumulation has been observed in *A. gossypii* cultures exposed to light, resulting in a 1.5-fold increase in RF production compared with that in cultures grown in the dark [178]. Likewise, low concentrations of the oxidative stress-inducing compounds H_2_O_2_, menadione, and α-tocopherol trigger RF overproduction in this fungus [174,175,176]. Notably, recent work by Park et al. has demonstrated the connections of RF overproduction with oxidative and ER stresses [86,179,180,200,201], as well as with the acetohydroxyacid synthase (AHAS) [213] and succinate dehydrogenase (SDH) activities [214].

Sirtuins are NAD^+^-dependent deacetylases involved in both prokaryotic and eukaryotic metabolism and stress responses. Disruption of sirtuin metabolism increases RF production by up to 4.3-fold [200]. Sirtuin-knockout mutants of *A. gossypii* show elevated acetylation of histone H3 lysine residues, resulting in thermosensitivity, sensitivity to genotoxic compounds, double-strand DNA breaks, genomic instability, chromosome loss, and altered antioxidant responses [215,216,217]. These mutants exhibit elevated ROS production, mitochondrial dysfunction, reduced mitochondrial membrane potential, and reduced expression of antioxidant genes, all of which favor RF biosynthesis. However, supplementing the growing culture medium of the fungus with N-acetyl-L-cysteine suppresses RF production, confirming that oxidative stress induced by sirtuin disruption is a central mechanism underlying RF overproduction.

Recently, the ER stressors tunicamycin, dithiothreitol, and cycloheximide were also demonstrated to increase RF production in *A. gossypii*. Adding tunicamycin or dithiothreitol to the growing culture medium of *A. gossypii* leads to increased intracellular ROS levels, elevated expression of RF biosynthetic genes, and RF overproduction; furthermore, dithiothreitol induces the expression of GR and SOD genes whereas tunicamycin does not. In contrast, the addition of cycloheximide induced apoptosis while simultaneously increasing RF production. Therefore, apoptosis triggered by ER stress and ROS generation may serve as a strategy to isolate novel RF-overexpressing *A. gossypii* strains [201].

Additionally, a 78–79% reduction in RF production was observed in *A. gossypii* by inhibiting succinate dehydrogenase (SDH) activity using malonate (a competitive SDH inhibitor) or the proteasome inhibitor MG-132 [214]. Similarly, the flavoprotein inhibitor diphenyleneiodonium decreased RF production by approximately 50%. Adding valine to the culture medium of *A. gossypii* MT strain induced a similar effect, attributed to the feedback inhibition of acetohydroxyacid synthase by valine, as this fungal strain exhibited elevated expression of this enzyme [213].

Overall, oxidative stress and regulation of antioxidant metabolism are important factors in RF overproduction by microorganisms, functioning as defense mechanisms against the stressful conditions generated by aerobic metabolism. This is consistent with the fact that aerobic conditions are required to achieve optimal RF production in yeasts [147,148,167,181,183,218], filamentous fungi [86,167,180,200], and *Bacillus subtilis*, the most widely used bacterium for industrial-scale RF production [158,168,219].

Ultrasound-stimulated cultivation [220] and adaptive laboratory evolution [221] are also promising approaches for enhancing RF production by *A. gossypii*.

Ultrasound-assisted cultivation of *A. gossypii* has been shown to enhance RF production by approximately 23.5% relative to that obtained with non-sonicated controls. This effect is mechanistically linked to ultrasound-induced oxidative stress, although excessive ultrasonic exposure may also cause irreversible physiological damage to microbial cells owing to acoustic cavitation. Acoustic cavitation occurs when gas microbubbles in an aqueous medium are exposed to an ultrasonic field and undergo rapid expansion, followed by violent collapse, generating extreme localized conditions, including intense shear forces, shock waves, transiently elevated temperatures, and localized exothermic energy release. These physicochemical effects promote sonolysis of water, subsequently leading to the formation of ROS, such as ·OH and H_2_O_2_ [222,223,224]. Ultrasound-stimulated cultivation of *A. gossypii* triggers both immediate and sustained intracellular H_2_O_2_ accumulation, causing oxidative stress in fungal cells, which is strongly associated with increased RF biosynthesis [220]. This observation is consistent with previous reports demonstrating that ultrasound-induced ROS production acts as a key regulatory signal influencing secondary metabolite biosynthesis and redox-related pathways in various microorganisms [224,225,226]. Collectively, these findings support the role of RF as an active redox metabolite that protects cells from oxidative damage.

An adaptive laboratory evolution (ALE) strategy was employed to successfully produce RF from cane molasses as a substrate by *A. gossypii*. Cane molasses contains high levels of furan aldehydes, phenolic compounds, and heavy metals that cause chronic oxidative stress and inhibit microbial metabolism and growth. However, prolonged adaptation to high molasses concentrations enabled *A. gossypii* to sustain and even enhance RF production. This adaptive phenotype correlates with an increased cellular capacity for free radical scavenging and improved the redox homeostasis acquired during the evolutionary adaptive process, reinforcing the central role of oxidative stress management in RF overproduction [221].

Taken together, ultrasound stimulation and ALE-based substrate adaptation converge on a common mechanistic framework, in which controlled oxidative stress exerts selective and regulatory pressures, promoting RF overproduction as part of a broader antioxidant and stress-mitigation strategy in *A. gossypii*.

Collectively, the available evidence suggests that oxidative stress should not be interpreted merely as a damaging condition, but rather as a regulatory signal that activates RF biosynthesis as part of a broader redox-balancing response. Therefore, future rational strain design strategies should not aim to maximize oxidative stress per se, but rather to finely modulate intracellular redox homeostasis. Controlled and phase-dependent redox perturbations—achieved through adaptive laboratory evolution, dynamic promoter engineering, oxygen supply regulation, or transient stress application—may enable activation of RF biosynthetic pathways while preserving cellular viability and metabolic robustness. In this context, redox-responsive regulatory networks represent promising targets for next-generation engineering approaches in RF biotechnology.

#### 5.1.2. The *Eremothecium ashbyi* Paradigm

Before the large-scale industrial production of RF was established, Guilliermond et al. discovered the flavinogenic potential of the filamentous fungus *Eremothecium ashbyi* in 1935 [100,167,227].

*A. gossypii* and *E. ashbyi* are closely related fungi [202,228,229], and the only natural RF overproducers within the genus *Eremothecium* [229]. They exhibit similar optimal conditions for the maximum specific RF production rates and RF yields, including pH, temperature, and substrate preference [228].

Despite these similarities, several differences exist in their RF biosynthetic behavior. In *A. gossypii*, glycine supplementation enhances RF production [150]. However, in *E. ashbyi*, glycine supplementation enhances biomass formation rather than RF production, whereas L-serine and L-threonine increase RF biosynthesis in this species [230,231,232]. Both species can utilize whey as a substrate for RF production, though *E. ashbyi* achieves higher RF yields from whey than those obtained with *A. gossypii* [228]. Interestingly, *E. ashbyi* produces significant amounts of extracellular FAD, whereas *A. gossypii* does not [233,234].

The major drawback of *E. ashbyi* is its tendency to lose its RF overproduction capability owing to genetic instability, pronounced sensitivity to freeze-drying and room-temperature storage, rapid reversion of mutant strains, and difficulty growing in inorganic salt–based media. Consequently, *E. ashbyi* has been gradually displaced from industrial RF production [100], though its use has not completely disappeared [71,72]. Under optimized cultivation conditions, reported RF titers range between 0.27 and 3.3 g L^−1^ [235,236,237], values significantly lower than those achieved by modern engineered *A. gossypii* strains.

Although the genetic instability and rapid phenotypic reversion of high RF-producing *E. ashbyi* strains are well documented [167,238,239], their molecular genetic basis remains poorly understood. Available evidence suggests that RF hyperproduction is associated with profound morphophysiological remodeling, including changes in hyphal architecture, lipid metabolism, and membrane fluidity, particularly during the stationary phase [240,241]. Such metabolic and structural alterations may impose a significant cellular burden, favoring the selection of revertants under non-selective storage conditions.

Notably, modern genome stability engineering approaches—such as chromosomal integration of deregulated biosynthetic genes, promoter refactoring, stabilization of regulatory circuits, or reinforcement of DNA repair systems—have not yet been systematically applied to *E. ashbyi* [17]. Elucidating the molecular determinants of instability and implementing rational genome stabilization strategies, therefore, represents a promising avenue to restore its industrial potential.

However, in recent years, several studies on the physiology, morphology, and vector expression systems of *E. ashbyi* have been published, highlighting its potential to develop strong and stable RF-producing strains through modern biotechnological approaches [237,242,243].

#### 5.1.3. Arcopilus aureus and Fusarium chlamydosporum as Novel Potential RF Producers

Recently, *Arcopilus aureus* was reported as an RF-producing fungus for the first time. This endophytic species, isolated from grapevine roots, forms a yellow pigment identified as a combination of RF and cochloroquinol II (another natural yellow pigment) based on via HPLC, FTIR, UV–Vis spectroscopy, and UPLC-DAD-ESI-QTOF-MS analyses. Approximately 0.213 mg kg^−1^ of the pigment was recovered from an ethyl acetate extract of the culture supernatant [244].

Further, proteomic studies of the opportunistic pathogenic fungus *Fusarium chlamydosporum* have revealed that several RFBP enzymes are upregulated in nitrogen-rich media [245].

However, further studies are required to fully assess the potential of *A. aureus* and *F. chlamydosporum* for RF production.

### 5.2. Yeasts as an Attractive Model for RF Biotechnology

Among various microorganisms described as RF producers, yeasts offer several biotechnological advantages, including ease of cultivation and handling, ease of genetic manipulation, homogeneous cell distribution in bioreactors, high specific growth rates, broad metabolic versatility, simple culture media formulations, and straightforward metabolite purification. Moreover, residual yeast biomass can serve as an alternative protein source [11,63,64,65,121,198,246].

#### 5.2.1. Flavinogenic Yeasts

Flavinogenic yeasts include *Meyerozyma guilliermondii*, *Candida famata* (teleomorph: *Debaryomyces hansenii*), *Candida albicans*, *Debaryomyces subglobosus* (anamorph: *Candida flareri*), and *Candida* (*Yamadazyma*) *membranifaciens*, all of which naturally produce RF [184,185,189,247,248].

These yeasts share three key characteristics: (1) they belong to the CTG or CUG clade, a group of yeasts that translate the CUG codon as serine—CTG(Ser1) or CUG(Ser1) in the Serinales order or CTG(Ser2) or CUG(Ser2) in the Ascoideales order—or as alanine in the CTG(Ala) or CUG(Ala) clade [247,248,249,250,251]; (2) they belong to the family Debaryomycetaceae and therefore to the Serinales order and the CTG(Ser1) clade; and (3) iron ions inhibit RF biosynthesis, whereas iron-deficient conditions strongly induce RF overproduction [17,167,181,182,183,187,189,248,252].

Iron-mediated inhibition of RF production has also been described in plants [17,253,254,255,256], and bacteria [17,167,257,258,259,260], but not in filamentous fungi [100,167]. Other metals, including copper, zinc, chromium, and cobalt, influence flavinogenesis to a lesser extent than iron [17,160,182,183,252,261,262,263,264]. The mechanisms underlying the iron-mediated inhibition of RF production by yeast remain unclear. However, extensive physiological and molecular work, primarily from the groups of D. V. Fedorovych, K. V. Dmytruk, Y. R. Boretsky, and A. A. Sibirny, has clarified the link between iron metabolism, RF overproduction, and oxidative stress in *M. guilliermondii* and *C. famata* [63,64,65,181,182,183,184,186,188,247,264,265].

Evolutionarily, plants, animals, and microorganisms display a strong metabolic interplay between RF and iron [266]. Fe^2+^ ions act as single-electron donors, while FMN and FAD participate in one- or two-electron transfer reactions, enabling broad redox functionalities as well as potential ROS formation and cellular damage [183,188,267,268]. In yeast cells, flavoproteins (FMN- and FAD-dependent enzymes) play key roles in iron uptake, ferric reduction [266], Fe–S cluster biosynthesis, and heme cofactor mobilization [269].

In flavinogenic yeasts, a strong regulatory relationship exists between iron acquisition and RF biosynthesis, wherein the proteins Sfu1, Sef1, Hap43, and Tup1 play a key role [17,63,64,65,187,189,247].

Sfu1, a transcription factor involved in iron homeostasis, negatively regulates the activities of Sef1 and Hap43. Sef1 functions as a transcriptional activator of iron uptake and transport genes and plays an important role in *Candida albicans* virulence; however, its activity is inhibited through direct physical interaction with Sfu1 under iron-replete conditions. Hap43 acts as a transcriptional repressor of iron assimilation genes under iron-limited conditions and is repressed by Sfu1 under iron abundance. Further, Tup1 serves as a global transcriptional corepressor involved in regulating morphological transitions and metabolic processes, including ferric reductase activity and ferrous iron transport [270,271,272,273,274].

Deletion of *SFU1* or *TUP1* (genes encoding the Sfu1 and Tup1 proteins, respectively) increases RF production and intracellular iron accumulation in *C. famata*, *M. guilliermondii*, and *C. albicans* [65,187,247]. In contrast, deletion of the *SEF1* gene leads to the loss of flavinogenesis and reduced intracellular iron accumulation, demonstrating its critical role in RF biosynthesis in the aforementioned yeasts [17,63,64,189,247]. Further, the global corepressors Tup1 and Hap43 cooperate in the flavinogenesis induced by low iron levels in *C. albicans* [187].

Notably, Sfu1 plays a critical role in the pathogenesis of *Candida albicans*. Under iron-rich conditions such as those found in the bloodstream of mammalian hosts, Sfu1 represses the activities of Hap43 and Sef1, thereby attenuating iron uptake and preventing the harmful effects of excessive iron accumulation, including the oxidative damage mediated by Fenton chemistry [187,189,272].

This coordinated regulation of iron metabolism and RF biosynthesis has been identified as a potential target for antifungal drug development against *C. albicans*, as inhibition of RF synthesis in this yeast species results in the loss of pathogenicity and severe growth defects [189]. In contrast, deregulation of iron homeostasis in the non-pathogenic yeast *Candida famata* represents a promising strategy for enhancing RF overproduction through metabolic engineering approaches [63,64,65]. However, the key aspects of regulatory networks linking iron metabolism and RF biosynthesis remain insufficiently characterized and warrant further investigation [63,64,65,247].

##### *Candida famata*: The Most Promising Flavinogenic Yeast

*C. famata* is the anamorph of *Debaryomyces hansenii* [185,275,276]. For decades, *C. famata* VKM Y-9 has served as the principal model for constructing RF-overproducing yeast strains [102,130,136,198,277] and elucidating RF regulatory mechanisms [63,64,65].

Taxonomic reassessment suggested that *C. famata* VKM Y-9 was an anamorph of *D. subglobosus* (anamorph: *Candida flareri*) [276]. However, to maintain consistency, the name *C. famata* is used here when referring to the wild-type strain VKM Y-9 and its derived mutant strains (AF-4, BRP, and BRPI).

Engineered *C. famata* strains can produce 16–20 g L^−1^ of RF under optimized fermentation conditions [6,67,87,136], representing a dramatic enhancement compared with wild-type strains, which typically produce only 1–3 mg L^−1^ [64,65]. Industrial RF production by this yeast species was discontinued by ADM Company (USA) approximately two decades ago because of its high sensitivity to iron leaching from steel equipment, genetic instability, and low economic yields [6,9,17,22,66,87,90].

Given the tight regulatory coupling between iron homeostasis and RF biosynthesis in *C. famata*, metabolic engineering of iron-responsive regulatory circuits represents a promising strategy to mitigate the inhibitory effects of iron leaching from industrial equipment [63,64,65]. Targeted modulation of transcription factors such as Sfu1 and Sef1, reinforcement of intracellular iron sequestration mechanisms, and refactoring of RF biosynthetic promoters may allow partial uncoupling of flavinogenesis from extracellular iron levels. Such strategies could enhance strain robustness and restore the industrial competitiveness of this yeast without requiring substantial modifications to bioreactor materials.

Nevertheless, *C. famata* remains one of the most extensively studied and promising flavinogenic species, with considerable potential for metabolic engineering to develop strong, stable, and competitive RF-overproducing strains. In this regard, RF production by *C. famata* has been enhanced via multiple complementary strategies. These include:

1. Overexpression of the RF excretase Rfe1, a yeast homolog of the mammalian BCRP/ABCG2 transporter, which facilitates RF efflux from the cell [277,278], and is responsible for RF transport into breast milk, blood, plasma, bile, and cerebrospinal fluid [3,279,280];
2. Overexpression of the *SEF1* gene under the control of a lactose-inducible promoter, enabling efficient utilization of whey as a carbon source [130,281];
3. Construction of strains overexpressing xylose reductase and xylitol dehydrogenase to achieve efficient RF production from sugarcane bagasse hydrolysate as a fermentation substrate [136];
4. Engineering of genes encoding PRPP synthase and PRPP amidotransferase for increased precursor supply to the RFBP [102];
5. Subsequent homologous overexpression of *RIB1* and *RIB6* genes, encoding GTP cyclohydrolase II and DHBP synthase, respectively [198]; and;
6. Coordinated co-overexpression of *RFE1* (RF excretase), *GND* (6-phosphogluconate dehydrogenase), and *RIB6* genes, resulting in enhanced RF titers when whey is used as the substrate [131].

##### *Meyerozyma guilliermondii*: A Model Yeast for Flavinogenic Research

*M. guilliermondii* (basionym: *Pichia guilliermondii*; heterotypic synonym: *Candida guilliermondii*) is a heterothallic, Crabtree-negative yeast incapable of growing anaerobically. It is part of the human microbiota and is typically harmless; however, it can occasionally act as an opportunistic pathogen in immunocompromised individuals. It is biotechnologically relevant because of its ability to metabolize hydrocarbons, produce xylitol, and secrete lipases [184,247,282,283]. *M. guilliermondii* serves primarily as a model organism for studying RF biosynthesis, transport, enzyme function, and gene regulation in yeasts [69,184] rather than as an industrial production host. Nevertheless, under optimized culture conditions, wild-type strains can produce approximately 23–34 mg L^−1^ of RF [199], reflecting moderate flavinogenic potential.

Mutant strains of *M. guilliermondii*, specifically rib80-1018-31, rib81-131-6, and hit1-1, show derepressed RF biosynthetic enzymes and enhanced iron uptake, leading to constitutive RF overproduction and intracellular iron accumulation compared with those in the wild-type strain, which overproduces RF only under iron-deficient conditions [181,183,188].

Wild-type and mutant strains of *M. guilliermondii* also overproduce RF and malondialdehyde (MDA) under exposure to oxidative agents such as methylviologen, menadione, CdCl_2_, or H_2_O_2_, with mutants showing increased sensitivity [181].

Under iron-deficient conditions (~0.18 μM Fe^2+^) combined with cobalt excess (9 mM CoCl_2_), yeast cells exhibit dysregulated iron homeostasis accompanied by increased oxidative stress, leading to elevated production of RF, ROS, and MDA [183]. Co^2+^ ions compete with Fe^2+^ at the transport and regulation level, increasing oxidative stress and, in response, stimulating iron absorption systems and promoting RF biosynthesis, while affecting the activities of antioxidant enzymes (CAT and SOD) and GSH levels [207,284,285].

Furthermore, ROS production increased by several orders of magnitude upon eliminating the genes involved in GSH biosynthesis compared with those in the wild-type strain, highlighting the close functional relationship between ROS biosynthesis and cellular redox homeostasis [265].

Overall, both iron limitation and iron hyperaccumulation generate oxidative stress conditions that activate RF biosynthesis, likely through redox-dependent regulatory mechanisms affecting the RFBP, thereby reinforcing the role of RF as a key component of the cellular antioxidant response [17,183,184,188,268].

##### *Debaryomyces* *hansenii*

RF production by *D. hansenii* var. *hansenii* MTCC 3574 has been explored using ultrasound stimulation during late exponential growth (60–72 h of incubation), yielding up to 240 mg L^−1^ RF with minimal cell damage [286]. As ultrasound promotes ROS generation [220,222,223,224], the observed RF enhancement is likely attributable to oxidative stress. *D. hansenii* NRRL Y-7426 and its isogenic *Dhhog1*Δ mutant (*HOG*::*SAT1*-yeYFP1) have revealed, for the first time, a connection between the mitogen-activated protein (MAP) kinase Hog1, which is central to the osmotic stress response, and RF biosynthesis [287].

##### *Yamadazyma* *membranifaciens*

*Y. membranifaciens*, formerly classified as *Candida membranifaciens*, is the most recently described flavinogenic yeast. It belongs to the CUG(Ser1) clade (Serinales order), Debaryomycetaceae family, and the *Yamadazyma* genus [248,288].

Four *Y. membranifaciens* strains (IST 495, IST 498, IST 507, and IST 626) were isolated in Portugal from a soil sample, of which strain IST 626 showed the highest RF production (~125 mg L^−1^) in 225 h of cultivation in YNB medium using xylose as the carbon source and with glycine supplementation under iron-free conditions. RF production decreased to ~6 mg L^−1^ when 2.0 μM FeCl_3_ was added [248].

Earlier work on *Y. membranifaciens* subsp. *flavinogenie* W14-3, isolated from seawater in the Eastern China Sea, showed that RF production occurs only under strictly aerobic conditions [289].

#### 5.2.2. Other RF-Producing Yeasts

##### *Candida tropicalis* and *Schwanniomyces occidentalis*

These yeasts are interesting RF producers, although they lack one or more of the defining features of flavinogenic yeasts. Both belong to the Debaryomycetaceae family, but only *C. tropicalis* belongs to the CUG clade [249]. *S. occidentalis* likely does not belong to the CUG clade [290,291].

Both yeast species overproduce RF under iron-deficient conditions and suppress RF production under excess iron [17,159,182,184]. However, some *C. tropicalis* strains do not produce RF at all [63,64,292].

Furthermore, recent studies on the regulatory mechanisms of Sef1 in *C. famata* and *C. tropicalis* have revealed notable differences in their flavinogenic potential, highlighting the distinctive regulatory features of *C. tropicalis* [63,64].

As mentioned above, deletion of *SEF1* in *C. famata* resulted in complete loss of flavinogenesis. However, when the promoter region of *SEF1* from *C. tropicalis* was fused with the open reading frame (ORF) of the *SEF1* gene and heterologously expressed in *SEF1*-deficient *C. famata* strains, their flavinogenic capacity was partially restored. Notably, this restoration was less pronounced than that achieved using the *SEF1* promoter from *Candida albicans*. Based on these observations, *C. tropicalis* is proposed to represent an intermediate evolutionary state between flavinogenic and non-flavinogenic yeasts, reflecting the transitional regulatory architecture of RF biosynthesis [64].

##### *Hyphopichia* *wangnamkhiaoensis*

*H. wangnamkhiaoensis* (formerly known as *Candida wangnamkhiaoensis* and *Wickerhamia* sp. X-Fep) is an amylolytic [246,293,294], lipolytic [295], and oleaginous yeast species [296] recently identified as an RF producer.

During α-amylase production studies, a yellow extracellular pigment was detected in the yeast growing medium [297,298]. This pigment was separated by RP-HPLC and identified as RF using UV–Vis, fluorescence, and ^1^H NMR spectroscopy [299].

*H. wangnamkhiaoensis* exhibits efficient RF production when grown on glucose as the carbon source, ammonium sulfate as a nitrogen source, and supplemented with biotin and glycine at an aeration rate of 1 vvm in a bubble column bioreactor–conditions that collectively favor RF biosynthesis. Notably, the maximum RF production occurred at 18–24 h of incubation, a fermentation time significantly shorter than that of the other yeasts [121]. Single-stage continuous culture studies have demonstrated a 149% increase in specific RF productivity compared with that of batch culture [152].

Linares-Martínez [297] reported that the RF produced by *H. wangnamkhiaoensis* initially accumulated intracellularly, predominantly during the first six hours of incubation. As the culture progressed to the late exponential (24 h) and stationary (30 h) growth phases, the RF produced was entirely excreted into the extracellular medium.

**Figure 3:**
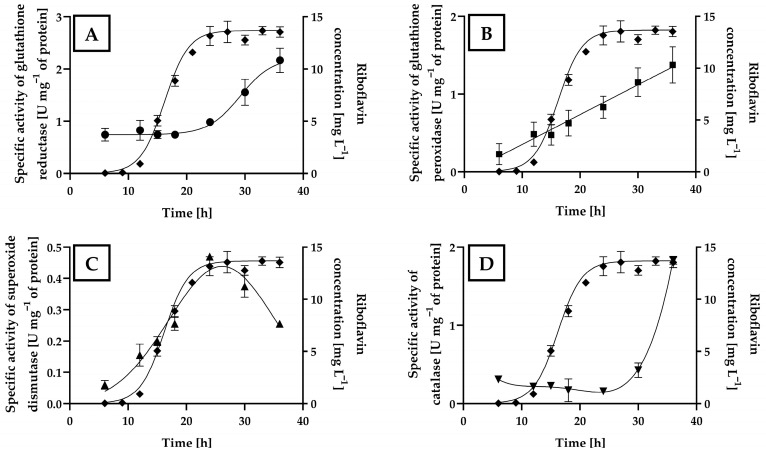
Variation profiles of the specific activities of antioxidant enzymes in *H. wangnamkhiaoensis* during RF production in batch culture using a bubble column bioreactor. (**A**) ● Specific activity of GR; (**B**) ■, specific activity of GPx; (**C**) ▲, specific activity of SOD; (**D**) ▼, specific activity of CAT; ♦, RF concentration. One unit (U) of GR and GPx activities was defined as the decrease in NADPH absorbance at 340 nm per minute at pH 7.0 and 25 °C. One unit (U) of CAT activity was defined as the decrease in H_2_O_2_ absorbance at 240 nm per minute at pH 7.0 and 25 °C. One unit (U) of SOD activity was defined as the inhibition percentage of nitroblue tetrazolium at 560 nm per minute at pH 10.2 and 25 °C. This figure has been reproduced from the doctoral thesis of R. A. Jiménez-Nava [218] with the permission of the author and his thesis supervisors (Prof. Dr. E. Cristiani-Urbina and Prof. Dr. G. M. Chávez-Camarillo). For detailed information on the experimental procedures, please refer to the cited work.

Subsequent research on this yeast strain revealed that during the initial incubation period (6–15 h) when RF accumulates intracellularly, the specific activities of glutathione peroxidase (GPx), GR, SOD, and CAT are relatively low [218]. However, once RF is excreted into the extracellular medium, the specific activities of GR, CAT, GPx, and SOD increase markedly, reaching levels approximately 2, 5, 5, and 7 times higher, respectively (Figure 3) [218].

*H. wangnamkhiaoensis* is thought to produce RF as a defense mechanism against the oxidative stress arising during aerobic cultivation in a bubble column bioreactor with an air flow of 1 vvm. As RF is oxidized, it becomes toxic to cells, prompting its excretion into the extracellular medium. Once RF is excreted by the cells, the yeast must activate alternative antioxidant systems, such as GR, GPx, CAT, and SOD, to reduce oxidative stress and restore redox balance [218]. Therefore, RF production by *H. wangnamkhiaoensis* appears to be closely associated with antioxidant metabolism.

Currently, whether *H. wangnamkhiaoensis* fully conforms to the previously proposed definition of flavinogenic yeasts remains unclear. Taxonomically, *H. wangnamkhiaoensis* belongs to the order Serinales and family Debaryomycetaceae [300]. However, as mentioned above, not all species in this family have been unequivocally recognized as yeasts of the CUG clade [290,291]; therefore, the inclusion of *H. wangnamkhiaoensis* within the CUG clade remains unresolved.

Furthermore, previous studies have indicated that RF biosynthesis is negatively affected by Fe^3+^ ions. When cultivated in YNB medium containing approximately 0.2 mg L^−1^ of FeCl_3_, this yeast strain produces 9.62 ± 0.49 mg L^−1^ of RF [218,298]. In contrast, supplementation with 2 mg L^−1^ FeCl_3_ results in drastically reduced RF production (0.49 ± 0.12 mg L^−1^) [298]. Overall, despite its potential, additional studies are required to elucidate the regulatory mechanisms governing RF biosynthesis in this yeast species.

##### *Candida hispaniensis*, *Rhodotorula glutinis*, and *Yarrowia lipolytica*

Non-flavinogenic yeasts have also received considerable attention as alternative RF producers. Using a Plackett–Burman experimental design, lactose, KH_2_PO_4_, and agitation rate were identified as key factors enhancing RF synthesis in *R. glutinis*, reaching 88.25 mg L^−1^ [301].

In another approach, mating between an RF-overproducing mutant and a xylose-utilizing mutant of *Y. lipolytica* yielded a diploid strain capable of producing RF from either xylose (97 mg L^−1^) or glucose (102 mg L^−1^) as carbon and energy sources, and both presented higher titers than those achieved by the parental strains grown on the same substrates [135].

More recently, *C. hispaniensis* CBS 99961^T^ was reported to produce up to 34.6 mg L^−1^ of RF after ~120 h of cultivation in YNB medium using glucose as the carbon source [302].

### 5.3. RF Production and Its Regulation in Bacteria

As previously mentioned, in bacteria, the *rib* operon riboswitch (*RFN* element) constitutes the principal regulatory mechanism controlling both RF biosynthesis and the expression of the RF transporter (RibU) in organisms incapable of biosynthesizing the vitamin de novo [6,11,17,22,69,85,90,91,190,191,192,193,194].

Further, the RibR protein can override this riboswitch-mediated regulation, enabling continuous expression of the *rib* operon and RibU transporter. RibR expression is induced by methionine or taurine and repressed by MgSO_4_, suggesting a regulatory interdependence between sulfur metabolism and RF biosynthesis [190,303].

Riboswitches are noncoding RNA structural domains generally located in the 5′ untranslated region (5′-UTR) of bacterial mRNAs. Their function is to detect and bind directly to small molecules or ions (ligands), thereby exerting cis-regulatory control, either at the transcriptional or translational level over genes involved in the biosynthesis, transport, or utilization of ligands or related metabolites [304,305,306,307].

Structurally, riboswitches comprise two domains: (1) the aptamer domain (highly conserved), which is responsible for ligand binding through ion-ion or hydrogen bonds, stacking interactions, or molecular packing, and (2) the expression platform (less conserved), which undergoes conformational rearrangements upon ligand binding, leading to the termination of transcription, blocking of translation initiation, or other regulatory outcomes. Therefore, riboswitches function as molecular ON/OFF switches, adjusting gene or operon expression (repression or activation) in response to intracellular ligand concentration [305,306,307,308].

The major classes of riboswitch ligands include coenzymes (FMN, thiamine pyrophosphate, cyanocobalamin, tetrahydrofolate, NAD^+^), carbohydrates (PRPP, glucosamine-6-phosphate), purines (adenine, guanine, xanthine), amino acids (L-glutamate, L-lysine, glycine), and ions (F^−^, Mg^2+^, Ni^2+^, Co^2+^, Fe^2+^, Mn^2+^) [304,307,308,309].

Within this regulatory landscape, the *RFN* element is distinguished by its specific binding to FMN, which modulates the transcription of RF biosynthetic genes [190,192,308]. However, this description is accurate only for Gram-positive bacteria, in which RF biosynthetic genes are arranged in a single operon, and the *RFN* element resides upstream of the first gene, acting through transcriptional attenuation. In contrast, in most Gram-negative bacteria, RF biosynthetic genes are dispersed throughout the chromosome, and only the *ribB* (encoding DHBP synthase) and *ribH2* (encoding lumazine synthase) genes are generally subject to *RFN*-mediated control, mainly at the translational initiation level, though transcriptional regulation can also occur [192,308,310,311].

**Figure 4:**
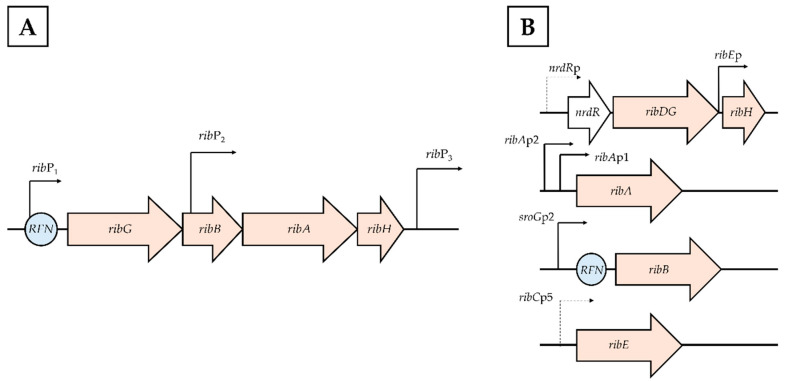
Schematic representation and nomenclature of the typical arrangement of RF biosynthetic genes in Gram-positive and Gram-negative bacteria. (**A**) Organization of the *rib* operon in *Bacillus subtilis* and (**B**) genomic arrangement of the RF biosynthetic genes in *Escherichia coli*. Light pink indicates structural genes; light blue indicates the *RFN* element; and white indicates adjacent non–RF biosynthetic genes in *E. coli*. Solid arrows represent confirmed promoters, whereas dashed arrows indicate predicted promoter regions. The figure was constructed based on information reported in references [11,17,69,90,311].

The organization of RF biosynthetic genes in a representative Gram-positive bacterium (*B. subtilis*) and Gram-negative bacterium (*E. coli*) is shown in Figure 4.

The classical nomenclature for the RF biosynthetic genes in *B. subtilis*, listed in the order in which the enzymatic reactions occur, is as follows: *ribA* (bifunctional GTP cyclohydrolase II/DHBP synthase), *ribG* (bifunctional DARPP deaminase/ARPP reductase), *ribH* (lumazine synthase), and *ribB* (RF synthetase). In *E. coli*, the nomenclature, listed according to the order of enzymatic reactions, is: *ribA* (monofunctional GTP cyclohydrolase II), *ribB* (monofunctional DHBP synthase), *ribDG* (bifunctional DARPP deaminase/ARPP reductase), *ribH* (lumazine synthase), and *ribE* (RF synthetase) [6,17,303,310,311].

Additionally, in bacteria, FMN and FAD biosynthesis is catalyzed by a bifunctional RF kinase/FAD synthetase encoded by the *ribC* gene in Gram-positive bacteria and the *ribF* gene in Gram-negative bacteria [17,303,311].

Notably, an *RFN* element upstream of the *ribH2* gene (a paralog of the *ribH* gene) has been identified only in a limited number of bacterial species, including *Brucella melitensis*, *Mesorhizobium loti*, *Pseudomonas fluorescens*, *P. syringae*, *Rhodopseudomonas palustris*, and *Sinorhizobium meliloti* [310].

#### 5.3.1. *B. subtilis*, the Workhorse of Microbial RF Production

*B. subtilis* is not naturally a high RF producer. Wild-type strains typically produce only 0.02 –4 mg L^−1^ of RF [6,17,312,313]. However, classical mutation breeding, strain selection, fermentation optimization, and advanced metabolic engineering have dramatically enhanced RF production, yielding titers ranging from 0.726 to 29 g L^−1^ in engineered strains [20,66,70,88,92], positioning this species among the most competitive industrial producers.

Notably, much of the current information on microbial RF biosynthesis, transport, and metabolic regulation has been elucidated using *A. gossypii* and *B. subtilis* as model organisms [22]. Several of the highest RF-producing microbial strains developed to date have been derived from *B. subtilis*.

Compared with fungal RF production, which typically requires 6–8 days of incubation to reach peak titers, *B. subtilis* reaches its maximum production within 2–3 days, with high yields even in simple fermentation media [92]. Therefore, *B. subtilis* is considered one of the most efficient and versatile chassis organisms for RF biosynthesis.

A broad range of strategies have been employed to enhance RF production in *B. subtilis*, from classical UV mutagenesis [314] to CRISPR–Cas9 genome editing and rational engineering of riboswitches governing purine and RF homeostasis [190].

Recently, Zhang et al. constructed *B. subtilis* S24, one of the strongest flavinogenic strains known to date, which produces 29 g L^−1^ of RF in only 52 h of fed-batch cultivation (Table 1). This remarkable RF titer was achieved using a phase-dependent promoter to overexpress the *rib* operon and by replacing the native bifunctional GTP cyclohydrolase II/DHBP synthase with a monofunctional DHBP synthase from *E. coli*. Phase-dependent promoters are expressed exclusively during the late logarithmic and stationary phases of growth; therefore, driving *rib* operon expression using such promoters prevents the plasmid instability induced by the accumulation of toxic intermediates, such as ArP [88].

Additional investigations from diverse research groups have contributed toward elucidating the complex interrelationships among the RFBP, PPP, and purine networks, including the biosynthesis, degradation, interconversion, and salvage pathways, in *B. subtilis*. This knowledge is essential for the rational design of next-generation RF overproducing strains.

As noted earlier, pyrimidine and adenosine metabolism compete with guanosine metabolism [70,95,127]. Thus, downregulation or deletion of the enzymes involved in adenosine and pyrimidine metabolism has been effective in enhancing RF biosynthesis [126,127,134]. Conversely, upregulating enzymes associated with RF biosynthesis, guanosine metabolism, or redirecting carbon flux within the PPP has also proven to be a successful strategy for improving RF production in *B. subtilis* [70,88,126,132,134,137].

As previously mentioned, oxidative metabolism significantly affects RF biosynthesis. Recently, hypoxic conditions were found to severely compromise purine and nitrogen metabolism in the *B. subtilis* RF1 strain, thereby limiting RF production. To overcome these limitations, the engineered *B. subtilis* aPaGaTgV strain expresses the *vgb* gene encoding *Vitreoscilla* hemoglobin and shows inhibited translation of the *glnR* and *tnrA* transcriptional regulators, which control ammonium assimilation and intracellular nitrogen metabolism, respectively. The combined effect of these modifications enables RF production to reach 10.71 g L^−1^ in fed-batch culture [158].

Another recent finding concerns the roles of biofilm biosynthesis and isocitrate dehydrogenase activity in an RF-overproducing *B. subtilis* U3 strain isolated through atmospheric and room-temperature plasma (ARTP) mutagenesis coupled with droplet-based microfluidic screening. However, the synergistic effects of mutations that influence biofilm formation and isocitrate dehydrogenase expression remain unclear. Furthermore, analysis of the exhaust gas composition during fed-batch fermentation revealed a reduced carbon flux toward the tricarboxylic acid cycle [157].

The most relevant and recent approaches for improving RF production by *B. subtilis* are summarized in Table 1.

#### 5.3.2. *Escherichia coli*

*E. coli*, particularly strains derived from nonpathogenic *E. coli* K-12 and *E. coli* B, is a cornerstone model organism for biotechnology, genetics, and metabolic engineering owing to its well-defined genetic background, high specific growth rate, short doubling time, and a broad array of advanced molecular tools [11,66,140,149,315]

Similar to *B. subtilis*, *E. coli* does not naturally produce high levels of RF. Under optimal culture conditions, the *E. coli* BL21(DE3) strain can produce approximately 85 mg L^−1^ of RF owing to a point mutation in the *ribF* gene encoding the bifunctional flavokinase/FAD synthetase, which promotes RF accumulation [315]. Through multiple metabolic engineering strategies, RF-overproducing strains have been developed, achieving titers ranging from 0.388 to 10.4 g L^−1^ [95,140,149,156,316].

For instance, by substituting the native *ribB* promoter with the constitutive *tac* promoter in the RF01 strain (derived from *E. coli* K-12), together with overexpression of guanylate kinase, nucleoside-diphosphate kinase, PRPP amidotransferase, PRPP synthase, and a mutation in the *purA* gene (codifying for the adenylosuccinate synthase) to attenuate AMP synthesis from IMP, enhance GTP synthesis, and relieve feedback inhibition in the PBP, the engineered *E. coli* RF18S strain was obtained, which produced 388 mg L^−1^ of RF [140].

The *E. coli* RF05S-M40 strain could produce 2.7 g L^−1^ of RF in approximately 60 h of flask cultivation in an optimized culture medium. This strain was constructed by inserting multiple copies of a synthetic operon carrying the native RF-biosynthetic genes of *E. coli*; disrupting the *pgi*, *edd*, and *eda* genes, which encode glucose-6-phosphate isomerase, phosphogluconate dehydratase, and 2-keto-4-hydroxyglutarate/2-dehydro-3-deoxy-phosphogluconate aldolase, respectively, to enhance carbon flux through the PPP, decrease glycolytic flux, and block the Entner–Doudoroff pathway (EDP); overexpressing acetyl-CoA synthetase to reduce acetate production; and introducing a weaker RBS upstream of *ribF* to limit the conversion of RF to FMN and FAD [149].

However, the RF05S-M40 strain requires isopropyl β-D-1-thiogalactopyranoside (IPTG) to induce expression of the plasmid-encoded synthetic *rib* operon; the plasmid itself exhibits high instability under varying culture conditions, which is an undesirable feature for industrial application. To overcome these drawbacks, a highly stable artificial RF operon was designed and expressed in *E. coli* K-12 MG1655, yielding the RF03T strain, which exhibited a 22% plasmid-loss rate at 23 h compared with 93% in the RF05S-M40 strain under the same conditions, and enhanced RF production. Further deletion of 6-phosphofructokinase I and additional blockage of the EDP led to generation of the LS02T strain, capable of producing 667 mg L^−1^ RF in flask culture and up to 10.4 g L^−1^ in a 71 h fed-batch fermentation process [156].

**Table 1:** Molecular strategies employed to enhance RF production by *B. subtilis.*

Parental Strain (Resulting Strain)	Manipulated Genes ^a^	Remarks	Maximum RF Titer Obtained, Fermentation Time, and Fermentation Mode Used	Reference
*B. subtilis* BSR (*B. subtilis* BEX5)	*guaB* + *guaA* + *gmk* + *ndk* + *ribA* +	Coordinated arrangement of diverse genes encoding enzymes catalyzing bottleneck reactions in the conversion of IMP to DARPP using the synthetic plasmid pEX5.	726 mg L^−1^; 48 h; batch culture in flask.	[70]
*B. subtilis* LY (*B. subtilis* BSR04)	*rib* operon + *purA* * rpe * gnd *	Enhanced pentose metabolism, reduced biosynthesis of adenosine intermediates, and deregulated RF pathway.	977.3 mg L^−1^; 60 h; batch culture in flask.	[126]
*B. subtilis* RF (*B. subtilis* RF01)	*gntP* +	Overexpression of the gene encoding gluconate permease enabled efficient use of sodium gluconate as a carbon source for RF production.	1.44 g L^−1^; 12 h; batch culture in a 7 L mechanically agitated bioreactor.	[137]
*B. subtilis* BEX5 (*B. subtilis* BSRE4/pMX45)	Δ*apt* *perR* * tkt *	Reduced AMP synthesis through the salvage pathway, suppression of deoxynucleotide synthesis to enhance carbon flux towards GTP synthesis.	3.47 g L^−1^; 72 h; batch culture in flask.	[70]
*B. subtilis* LR13 (*B. subtilis* RX22)	*pyrE* – *pyr* operon – *ykgB* + Δ*codY*	Efficient use of urea as a nitrogen source, elevated carbon flux towards PRPP, and diminished biosynthesis of pyrimidine intermediates.	7.01 g L^−1^; 72 h; batch culture in flask.	[134]
*B. subtilis* RF1 (*B. subtilis* aPaGaTgV	*vgb* + *glnR* – *tnrA* –	Expression of bacterial hemoglobin from *Vitreoscilla*, enhanced ammonium assimilation, and intracellular nitrogen metabolism to overcome hypoxia-induced metabolic defects.	10.71 g L^−1^; 48 h; fed-batch fermentation in a 5 L mechanically agitated bioreactor.	[158]
*B. subtilis* RF1 (*B. subtilis* RF1-L3)	*zwf* + *ribA* + *ywlF* +	Enhanced carbon flux towards pentose and purine biosynthesis by multiple gene expression using a tunable intergenic region (TIGR) library from *E. coli*.	11.77 g L^−1^; 48 h; fed-batch fermentation in a 5 L mechanically agitated bioreactor.	[132]
*B. subtilis* S1 (*B. subtilis* U3)	*sinR* * icd *	Mutagenesis through atmospheric and room-temperature plasma and droplet-mediated microfluidic screening of a mutant strain with mutations in isocitrate dehydrogenase and biofilm metabolism, and carbon flux redistribution in the TCA cycle.	24.3 g L^−1^; 46 h; fed-batch fermentation in a 7.5 L mechanically agitated bioreactor.	[157]
*B. subtilis* S1 (*B. subtilis* S24)	*ribB* + *rib* operon +	Overexpression of a deregulated *rib* operon and replacement of the *ribA* gene encoding a bifunctional GTP cyclohydrolase II/DHBP synthase with *ribB* gene encoding a monofunctional DHBP synthase from *E. coli*, using the phase-dependent promoter PyqgZ, only expressed in the late-logarithmic and stationary growth phases.	29 g L^−1^; 52 h; fed-batch fermentation in a 7.5 L mechanically agitated bioreactor.	[88]

^a^ “*” indicates gene mutation, “+” indicates overexpression, “–“ indicates downregulation, and “Δ” denotes gene knockout. Gene designations (with the encoded enzyme/protein in parentheses) are as follows: *apt* (adenine phosphoribosyltransferase); *codY* (CodY transcriptional regulator); *gnd* (6-phosphogluconate dehydrogenase); *guaA* (guanosine monophosphate synthase); *guaB* (inosine-5′-monophosphate dehydrogenase); *glnR* (GlnR transcriptional regulator); *gmk* (guanylate kinase); *gntP* (gluconate permease); *icd* (isocitrate dehydrogenase); *ndk* (nucleoside-diphosphate kinase); *perR* (PerR global regulator); *purA* (adenylosuccinate synthase); *pyrE* (orotate phosphoribosyltransferase); *pyr* operon (pyrimidine biosynthetic operon); *ribA* (bifunctional GTP cyclohydrolase II/DHBP synthase in *B. subtilis*); *ribB* (monofunctional DHBP synthase in *E. coli*); *rib* operon (RF biosynthetic operon); *rpe* (ribulose-5-phosphate 3-epimerase); *sinR* (SinR regulator); *tkt* (transketolase); *tnrA* (TnrA transcriptional regulator); *vgb* (*Vitreoscilla* hemoglobin); *ykgB* (6-phosphogluconate-1,5-lactonase); *ywlF* (ribose-5-phosphate isomerase); and *zwf* (glucose-6-phosphate dehydrogenase).

Interestingly, *E. coli* is one of the few bacterial species for which strong evidence of ROS-induced RF overproduction is available. In this bacterium, *ribA* expression is induced by the oxidative stress triggered by methylviologen, menadione, and plumbagin [317,318].

The principal studies aimed at improving RF biosynthesis in *E. coli* are summarized in Table 2 [95,140,149,156,316].

#### 5.3.3. Non-Conventional Flavinogenic Bacteria: The Forefront of RF Biotechnology

Advanced metabolic engineering approaches applied to *Corynebacterium glutamicum* have demonstrated its strong potential to produce up to 8.3 g L^−1^ RF [68,99,319]. In contrast, wild-type strains produce negligible quantities of the vitamin. These results underscore its emerging potential as an alternative industrial chassis. Heterologous expression of mannitol-catabolizing enzymes has enabled the use of predigested brown seaweed (*Laminaria hyperborea*), a microalga-derived substrate rich in glucose and mannitol [99]. Further, the construction of synthetic *rib* operons with high predicted translation initiation rates, combined with the overexpression of fructose-1,6-bisphosphatase and PRPP amidotransferase, has been shown to enhance RF production when using rice husk hydrolysate or spent sulfite liquor as substrates [68].

*Bacillus methanolicus* is another promising non-conventional chassis for RF production. A recent study demonstrated that the heterologous expression of a *rib* operon on a high-copy plasmid enabled RF production of approximately 523 mg L^−1^ using methanol as a carbon and energy source during fed-batch cultivation [98], representing a marked improvement over the wild-type strain, which produces only 0.01 mg L^−1^.

The thermophilic bacterium *Geobacillus thermoglucosidasius* has also emerged as a promising RF-producing organism [94]. A sophisticated strategy involving the heterologous overexpression of RF biosynthetic genes and upregulation of the precursor supply via PPP and PBP engineering has enabled a two-phase RF production system via dynamic catabolite-repression control. In this system, glucose is exclusively used for biomass formation, whereas RF biosynthesis is activated only during xylose catabolism. This artificial decoupling of trophic and productive phases allowed the production of up to 1.04 g L^−1^ RF in only 12 h of cultivation [94,96], whereas the wild-type strain does not produce detectable levels of RF.

Autotrophic bacteria also present unique opportunities for RF biotechnology. For instance, heterologous overexpression of RF biosynthetic genes in the photoautotrophic cyanobacterium *Synechococcus* sp. enabled the production of 28 mg L^−1^ RF over 38 days under dichromatic LED illumination, thereby preventing RF photolysis [93]. Conversely, homologous overexpression of the *ribA* gene in the chemolithoautotroph *Xanthobacter autotrophicus* enabled the synthesis of approximately 1.5 μg L^−1^ RF in an electrochemical cultivation system using the H_2_ generated by splitting water in an N_2_/CO_2_/O_2_ gas mixture as an energy source [97].

Although the RF production levels in these non-conventional hosts remain lower than those of industrial *A. gossypii* or *B. subtilis* strains, they offer several advantages such as thermophilic growth (*G. thermoglucosidasius*), use of low-cost (agro-industrial wastes or byproducts), non-food, or non-feed competitive substrates (*B. methanolicus*, *C. glutamicum*, *G. thermoglucosidasius*), and autotrophic growth on minimal media with almost non-organic nutrients (*Synechococcus* sp., *X. autotrophicus*). These advantages expand the future landscape of sustainable RF bioproduction [68,93,94,96,97,98,99,319].

**Table 2:** Molecular strategies for enhanced RF production in *Escherichia coli.*

Parental Strain (Resulting Strain)	Manipulated Genes ^a^	Remarks	Maximum RF Titer Obtained, Fermentation Time, and Fermentation Mode Used	References
*E. coli* RF01 (*E. coli* RF18S)	*ribB* + *gmk* + *ndk* + *purA* * purF *+ *prs* *+	Constitutive overexpression of the DHBP synthase, and overexpression of several PBP-related genes to attenuate AMP synthesis from IMP, enhance GTP synthesis, and diminish feed-back inhibition in the PBP.	388 mg L^−1^; 40 h; batch culture in flask.	[140]
*E. coli* BL21(DE3) (*E. coli* R6)	*ribF* –	Site-directed mutagenesis of the *ribF* gene using CRISPR/Cas9 technology to rationally diminish the activity of the RF kinase/FAD synthetase.	657 mg L^−1^; 72 h; batch culture in flask.	[316]
*E. coli* WY0T (*E. coli* WY40)	*ribF* – *purA* – *guaC* –	Fine-tuning the expression of three genes involved in metabolically competitive reactions using synthetic regulatory small RNAs to augment the carbon flux toward RF biosynthesis and diminish the synthesis of FMN and FAD.	1.45 g L^−1^; batch culture in flask.	[95]
*E. coli* K-12 MG1655 (*E. coli* RF05S-M40)	*ribA* + *ribB* + *ribD* + *ribH* + *ribE* + Δ*pgi* Δ*edd* Δ*eda* *acs* + *ribF* –	Overexpression of a synthetic/artificial *rib* operon in a high-copy plasmid; disruption of the glucose-6-phosphate isomerase and EDP to enhance carbon flux toward the PPP and diminish acetate production.	2.7 g L^−1^; 60 h; batch culture in flask.	[149]
*E. coli* RF03T (*E. coli* LS02T)	*ribA* + *ribB* + *ribD* + *ribH* + *ribE* + Δ*pfkA* Δ*edd* Δ*eda*	Overexpression of a synthetic/artificial *rib* operon in a highly stable, low-copy plasmid, disruption of 6-phosphofructokinase I and EDP to enhance carbon flux towards the PPP, and augmented biosynthesis of glycine.	10.4 g L^−1^; 71 h; fed-batch fermentation in a 5 L bioreactor.	[156]

^a^ ‘*’ indicates gene mutation, ‘+’ indicates overexpression, ‘–’ indicates downregulation, and ‘Δ’ indicates gene knockout. Gene designations (with the encoded enzyme/protein in parentheses) are as follows: *acs* (acetyl-CoA synthetase); *eda* (2-keto-4-hydroxyglutarate/2-dehydro-3-deoxy-phosphogluconate aldolase); *gmk* (guanylate kinase); *guaC* (GMP reductase); *ndk* (nucleoside-diphosphate kinase); *pfkA* (6-phosphofructokinase I); *pgi* (glucose-6-phosphate isomerase); *prs* (PRPP synthase); *purA* (adenylosuccinate synthase); *purF* (PRPP amidotransferase); *ribA* (GTP cyclohydrolase II); *ribB* (DHBP synthase); *ribD* (bifunctional DARPP deaminase/ARPP reductase); *ribH* (lumazine synthase); *ribE* (RF synthetase); *ribF* (bifunctional RF kinase/FAD synthetase).

#### 5.3.4. Novel RF-Producing Bacteria Isolated from Natural Sources

Over the last five years, several bacterial species have been newly identified as natural RF producers. These organisms have noteworthy physiological traits, ecological origins, and production capacities.

Recently, the acetic acid bacterium *Gluconobacter oxydans* FBFS97, isolated from a soil sample in China, was described for the first time as an RF producer. This bacterial strain preferentially uses fructose and tryptone as carbon and nitrogen sources, respectively, for RF production. Using a Plackett-Burman design, K_2_HPO_4_ and CaCl_2_ were identified as significant factors positively influencing RF production. The culture medium composition was further optimized using a central composite design, allowing RF production up to 23.24 mg L^−1^ [320].

RF production has also been documented in the bacterial strain *Novosphingobium panipatense* SR3 (MT002778), which was recently identified and isolated from Karish cheese [321]. Culture medium optimization using a statistical Box–Behnken design identified maltose, yeast extract, and glycine as the primary nutritional determinants for RF overproduction, enabling the strain to produce 497.12 mg L^−1^ of RF within 48 h of cultivation [322].

Additionally, six strains of lactic acid bacteria belonging to the *Weisella* and *Leuconostoc* genera, isolated from Spanish rye sourdoughs, were recently recognized as RF- and dextran-type exopolysaccharide-producers, with maximum RF production levels ranging between 203.5 and 684.5 μg L^−1^ [323]. Subsequent optimization efforts using one of the *Weisella* strains yielded two *Weisella cibaria* mutant strains with enhanced RF biosynthesis in two independent investigations. The strain BAL3C-5 B2 (also designated *W. cibaria* BAL3C-5 G15T) produced 3.45 mg L^−1^ of RF within 23 h [324], whereas strain BAL3C-5 C120T achieved 6.78 mg L^−1^ of RF within 16 h [325]. Although the parental BAL3C-5 strain demonstrated a strong potential for RF-enriched bread production [324], only the BAL3C-5 C120T strain was confirmed to be microbiologically safe for producing RF-fortified gluten-free bread [326] and oat-based fermented beverages [327].

In a recent bioprospecting study, *Citrobacter freundii* DHS18, *Delftia tsuruhatensis* DHS14, and *Enterobacter cloacae* DHS38, isolated from lamb kidney, spinach, and beef liver, respectively, were identified for the first time as RF producers, with production titers of 2.29, 1.45, and 2.48 mg L^−1^, respectively. Two additional isolates, *Lactobacillus plantarum* HDS27 and *Bacillus cereus* HDS07, also produced notable RF titers (3.69 and 2.9 mg L^−1^, respectively) [328].

The strain *Limosilactobacillus reuteri* AMBV339, isolated from a healthy female volunteer in the Isala Project (Belgium) [329], was recently recognized as an RF producer [330]. Strain AMBV339 could synthesize approximately 18.36 mg L^−1^ of RF in overnight culture in MRS medium. It effectively enriched fermented coconut beverages alone or in co-culture with *Streptococcus thermophilus* LMG18311, survived simulated gastrointestinal dialysis, did not disrupt the healthy human fecal microbiome, and selectively suppressed enteric pathogens [330].

Notably, in silico analyses of *L. reuteri* DSM 20,016 suggested the presence of putative RF biosynthetic genes [331], but, no RF production was observed in this strain.

Table 3 summarizes the emerging RF-producing bacteria, their sources of isolation, maximum RF titers, and distinguishing characteristics.

**Table 3:** Novel bacteria reported as natural RF producers.

Species	Isolation Source	Maximum RF Production (mg L^−1^)	Remarks	Reference
*Gluconobacter oxydans*	Soil	23.24	K_2_HPO_4_ and CaCl_2_ significantly affect RF production	[320]
*Leuconostoc falkbergense*	Rye sourdough	0.6845	Produces dextran exopolysaccharide	[323]
*Weisella cibaria*	Rye sourdough	0.5482	Produces dextran exopolysaccharide	[323]
*Novosphingobium panipatense*	Karish cheese	497.12	Maltose, yeast extract, and glycine are major effectors for RF production	[321,322]
*Citrobacter freundii*	Lamb kidney	2.29	Component of the healthy human gut microbiota	[328]
*Delftia tsuruhatensis*	Spinach	1.45	Opportunistic pathogen	[328]
*Enterobacter cloacae*	Beef liver	2.48	Opportunistic pathogen of nosocomial infections	[328]
*Limosilactobacillus reuteri*	Healthy female volunteer	18.36	Exhibits potential probiotic activity	[330]

## 6. Importance of RF Biosynthesis for Microbial Electron Transfer and Microbial Fuel Cells

A microbial fuel cell (MFC) is a bioelectrochemical system that converts the chemical energy of organic substrates into electrical energy through the anaerobic metabolism of electroactive microorganisms. During this process, electrons generated by microbial oxidation reactions are transferred from the cell surface to an extracellular electron acceptor, resulting in electric current generation. Extracellular electron transfer can occur via biological mediators such as *c*-type cytochromes, RF, and FMN or through exogenous conductive structures such as filaments or nanowires [133,332,333,334].

RF plays a central role in MFC development because it mediates electron transfer from outer-membrane cytochromes to the anode surface through cyclic redox reactions [133,335]. Moreover, exogenously supplied RF can significantly enhance electron exchange [336]. Engineering RF overproduction in electroactive microorganisms is an emerging strategy with substantial potential to improve MFC efficiency and support applications including wastewater treatment, pollutant biosensing, and the generation of renewable “green” electricity [133,332,333,334,337,338].

In the model electroactive bacterium *Shewanella oneidensis*, extracellular electron transfer is mediated by *c*-type cytochromes and RF. Fine-tuning the expression of genes encoding glucose-6-phosphate dehydrogenase, glycine hydroxymethyltransferase, and threonine aldolase increases the carbon flux toward RF biosynthesis, thereby enhancing extracellular electron transfer. Additionally, combined overexpression of the endogenous *c*-type cytochrome gene and fine-tuning of RF biosynthetic gene expression yielded a 3-fold increase in current density and a 2.34-fold increase in power density relative to that obtained with the parental strain [133].

A Cu^2+^ MFC biosensor based on an engineered RF-overproducing *E. coli* strain detects extracellular copper via RF-mediated increases in voltage output, representing a promising strategy for monitoring this highly polluting ion [337].

Furthermore, the RF produced and excreted by *Shewanella* sp. strain FDL-2 enhances the bioreduction of selenite [Se(IV)] and tellurite [Te(IV)], which are the most hazardous anthropogenic forms of selenium and tellurium, respectively. This strain bioreduced 10 mM Se(IV) and 5 mM Te(IV) within approximately 72 h. Genomic analysis revealed that RF synthetase genes were upregulated in the presence of these metalloids, supporting the role of RF in their detoxification [338].

## 7. Microbiological Production of FMN, FAD, Roseoflavin, and 8-Aminoriboflavin

RF has multiple natural structural analogs [339,340,341,342]. Of these, FMN and FAD, which are the biologically active forms of RF, are the most physiologically relevant.

**Figure 5:**
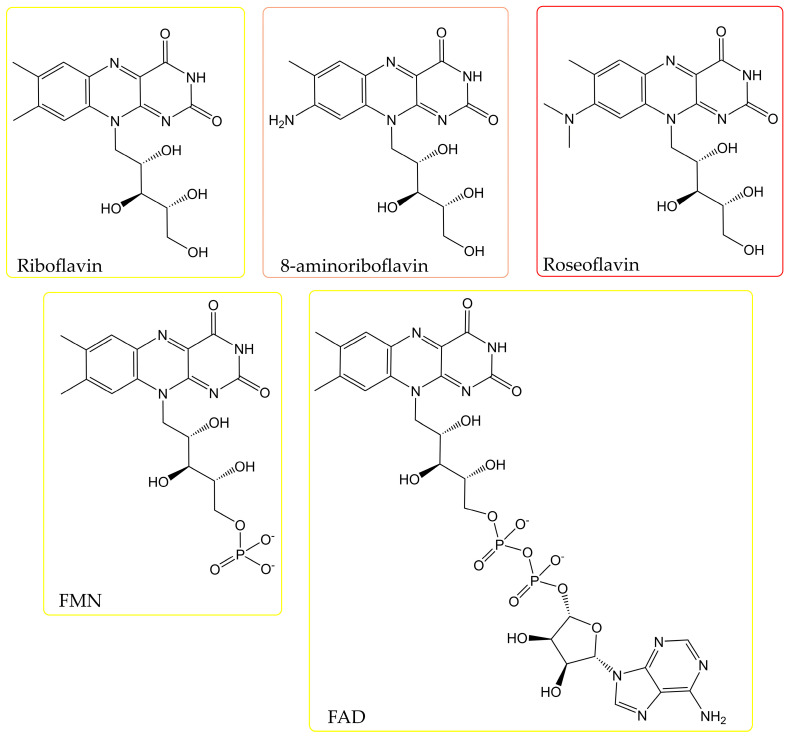
Comparison of the molecular structures of RF, its structural analogs (RoF and AF), and its derivatives (FMN and FAD). All structures were generated using PubChem database to ensure chemical accuracy and consistent graphical representation. The frame color matches the aqueous solution color of the molecule.

Recently, two additional natural RF analogs, roseoflavin (RoF) and 8-aminoriboflavin (AF), have attracted attention because of their antibiotic properties. These compounds are produced exclusively by *Streptomyces davaonensis* (heterotypic synonym: *Streptomyces davawensis*) and *S. cinnabarinus* [343,344,345,346,347,348]. Their chemical structures are shown in Figure 5.

FMN and FAD are produced either chemically or through chemo-enzymatic synthesis and are widely used as food additives or pharmaceuticals, owing to their markedly higher water solubility compared with that of RF (RF: 0.05–2.3 g L^−1^; FMN: 67 g L^−1^; FAD: 50 g L^−1^) [1,3,7,349,350]. However, these synthetic routes require high-purity chemicals (RF, FMN, adenine, and ATP) and enzymes (RF kinase, FAD synthetase, and FAD pyrophosphatase), resulting in extremely high production costs and low overall yields [9,67].

As previously mentioned, *E. ashbyi* is a natural producer of FAD, though several drawbacks limit its biotechnological applicability [6,11,17,100,233].

Historically, several bacterial species, including *Micrococcus luteus* (homotypic synonym: *Sarcina lutea*), *Corynebacterium ammoniagenes* (homotypic synonym: *Brevibacterium ammoniagenes*), and *E. coli*, have been proposed as biocatalysts for FMN and FAD biosynthesis using organic phosphate, adenine/ATP, and RF/FMN as precursors [351,352,353]. However, these approaches cannot be scaled to industrial levels owing to the high cost of the precursors and low yields [90].

Another major limitation of bacterial FAD production is that FMN represses flavin biosynthesis and transport pathways by binding to the *RFN* element, thereby preventing the overproduction of flavins (RF, FMN, and FAD) [17,67].

Genetic engineering methods have greatly improved FMN and FAD biosynthesis in *E. coli* and *C. famata* [90,233,234,354,355,356]. However, eukaryotic hosts are a more suitable chassis for FMN and FAD overproduction because, unlike most bacteria, RF kinase and FAD synthetase are encoded by separate genes (*FMN1* and *FAD1*) in eukaryotes. Bacteria typically harbor a single bifunctional enzyme encoded by *ribC* (Gram-positive bacteria) or *ribF* (Gram-negative bacteria), which catalyzes both FMN and FAD synthesis [9,67,303,311].

Owing to its extensive genetic background and well-characterized flavinogenic traits, *C. famata* has emerged as the most promising platform for the biotechnological production of FMN, FAD, RoF, and AF [67,233,234,349]. However, a recent study reported that *C. famata* produces detectable amounts of AF, but not RoF. In contrast, *Komagataella phaffii* produces both RoF and AF (22 and 130 mg L^−1^ of AF and RoF, respectively) [348].

The key aspects of FMN, FAD, AF, and RoF production by engineered *C. famata* strains are summarized in Table 4.

**Table 4:** Key aspects of FMN, FAD, AF, and RoF production by engineered *C. famata* strains.

Produced Compound	Highlights	Maximum Titer in mg L^−1^	References
FMN	Overexpression of the *FMN1* gene under the strong constitutive promoter *TEF1*	200–250	[233]
	Culture medium optimization through Plackett-Burman and Central Composite designs in a recombinant FMN-producing strain.	318	[67,357]
	Overexpression of the *SEF1* gene under the control of a lactose-induced promoter for the efficient use of whey as substrate.	540	[349]
FAD	Culture medium optimization using Plackett-Burman and Central Composite designs for the cultivation of a recombinant strain that overexpresses the *FMN1* and *FAD1* genes under the *TEF1* promoter.	452	[67,234]
AF	Heterologous expression of the *rosB* gene in an FMN-overproducing strain.	1.5	[348]
RoF	Heterologous expression of *FMN1*, *rosB*, *rosC*, and *rosA* in an RF-overproducing strain.	Not detectable	[348]

## 8. Perspectives and Concerns Surrounding RF Biotechnology

Among the diverse microorganisms described, *B. subtilis* and *A. gossypii* currently represent the most technologically mature and industrially competitive platforms for large-scale RF production, owing to their high titers, robust fermentation performance, and established regulatory acceptance. Emerging microbial systems, although promising, remain at developmental or application-specific stages.

RF biotechnology advanced substantially in recent decades, driven by developments in systems biology, synthetic biology, and metabolic engineering. However, despite the considerable achievements, regulatory, biosafety, and technological constraints continue to dictate the future trajectory of RF biotechnology.

In addition to regulatory and biosafety considerations, intrinsic metabolic and process-level limitations continue to define the economic boundaries of industrial RF production. Despite the remarkable advances in strain engineering and fermentation optimization, RF production by microbial fermentation still faces several integrated bottlenecks that ultimately determine its commercial competitiveness. From a metabolic perspective, the tightly regulated flux toward the immediate precursors GTP and Ru5P remains a central limitation, as these metabolites occupy key nodes in nucleotide and central carbon metabolism. Moreover, feedback regulation within the purine biosynthetic pathway restricts maximal precursor availability for RF synthesis [89].

Beyond pathway-level constraints, industrial performance is also influenced by oxygen transfer requirements [121,147,148,158,168,218,219], redox-balancing demands [180,181,183,200], genetic stability during prolonged cultivation [17,100], and downstream processing efficiency. Therefore, improving commercial competitiveness requires not only increasing volumetric productivity but also enhancing carbon yield efficiency, process robustness, and long-term strain stability under large-scale conditions.

Some challenges are particularly relevant in countries and regions of the world where strict legislation regulates the use of recombinant microorganisms in various applications such as food and feed [71,72,358,359,360,361,362,363,364].

One of the most notable concerns is the discovery of recombinant DNA and viable microbial cells in commercial batches of feed-grade RF (80%) [358,365]. This is particularly problematic in countries where the presence of recombinant DNA in food products is strictly prohibited. These events have raised concerns regarding the adequacy of containment strategies, robustness of downstream industrial processes, and the traceability of genetically modified strains in large-scale operations [22,199]. Although some RF products derived from *B. subtilis*, *A. gossypii*, and *E. ashbyi* have been evaluated as safe and compliant with EU FEEDAP guidelines [72,359,362,363,364], such incidents highlight the need for improved molecular surveillance strategies and quality assurance during industrial production.

The second primary concern is the potential development and spread of genes encoding antibiotic resistance. Conventional microbial strain engineering relies on antibiotic resistance markers for plasmid maintenance or selection, a practice that may facilitate the emergence or persistence of resistant microorganisms [221,366,367,368,369]. As RF-overproducing strains often require extensive genetic modifications, including pathway rewiring, promoter engineering, and multi-copy plasmid assemblies, antibiotic use during strain development and scaled fermentation is common [64,65,70,94,96,126,132,137,140,149,156,158,193,198,277,281,316,370,371]. The long-term clinical and ecological implications of releasing such microbial strains, even unintentionally, are a topic of active debate.

Several promising lines of innovation have been gaining interest in addressing these concerns. The use of non-genetically modified organisms (non-GMOs) obtained through classical mutagenesis, adaptive evolution, and high-throughput screening has attracted renewed interest [74,75,194,199,221,314].

Although these strains typically have lower RF volumetric productivity than that of their genetically modified counterparts, continuous improvements in atmospheric and room-temperature plasma (ARTP) mutagenesis, microfluidic screening, genome rearrangement, and evolutionary engineering provide increasingly effective alternatives that circumvent the regulatory restrictions on GMOs [157,316,372].

Second, the development of fully antibiotic-free selection and maintenance systems offers a robust strategy to eliminate reliance on antibiotic resistance markers. Emerging approaches include auxotrophic complementation, CRISPR-based counter-selection, toxin-antitoxin stabilization modules, and replicon partitioning systems, which have been successfully implemented in RF-producing strains of *B. subtilis* and *E. coli* [369]. These systems reduce biosafety risks, improve plasmid stability, reduce downstream purification costs, and facilitate regulatory approval.

The future is expected to usher in an accelerated integration of synthetic biology, genome-scale modeling, and machine-learning-guided metabolic design, transforming the next generation of RF-producing microorganisms. Advanced technologies, such as the increased use of unconventional hosts, dynamic regulation of metabolic pathways, modular cell engineering, and microbe-electrode hybrid systems—especially in situations where RF serves both as an electron carrier and a metabolic product—will likely broaden the applications of RF biotechnology beyond traditional use. Moreover, the rising demand for bio-based and sustainable processes aligns with global initiatives to valorize agro-industrial waste and by-products, minimize environmental impact, and develop cell factories that can operate with minimal inputs or under extreme conditions, and generate less polluting waste.

Overall, although challenges in biosafety and regulation raise significant concerns, the field is rapidly evolving toward more controllable, safer, and sustainable microbial platforms. Continued innovation coupled with stricter genomic surveillance, improved upstream and downstream process control, and global harmonization of regulations is expected to mitigate the current concerns and usher in a new era of safe, robust, and high-performance RF biotechnology.

## 9. Conclusions

RF biotechnology has advanced rapidly in recent decades and is driven by the integration of synthetic biology, metabolic engineering, and high-throughput screening technologies. However, several challenges remain unaddressed. Some countries and regions of the world have restrictions and strict scrutiny regarding the production and use of RF produced using genetically modified microorganisms. Detection of viable cells or recombinant DNA in commercial feed-grade products and reliance on antibiotic resistance markers for mutant selection have raised concerns regarding public and environmental safety. Consequently, the development of genetically stable microbial strains free of antibiotic resistance-encoding genes and complying with regulations is a key research priority. Current research indicates that a thorough understanding of RF biosynthesis regulation, metabolic flux distribution, and precursor availability is essential for designing high-yield strains.

Although the most productive microorganisms are currently *B. subtilis* and *A. gossypii*, exploring alternative organisms, including autotrophic, thermophilic, methylotrophic, and lactic acid bacteria, can offer new opportunities for industrial diversification and environmental sustainability. Furthermore, the use of RF as an active redox mediator in microbial electron transfer offers promising applications in pollutant biodetection, wastewater treatment, and renewable bioelectricity generation.

Overall, the future of RF biotechnology depends on balancing innovation with biosafety and sustainability to meet industrial, social, and environmental demands.

## Data Availability

No new data were created or analyzed in this study. Data sharing is not applicable to this article.
